# Overexpressed nicotinamide N‑methyltransferase in endometrial stromal cells induced by macrophages and estradiol contributes to cell proliferation in endometriosis

**DOI:** 10.1038/s41420-024-02229-3

**Published:** 2024-11-03

**Authors:** Shuhui Hou, Hui Xu, Shating Lei, Dong Zhao

**Affiliations:** 1 Department of Obstetrics and Gynecology, Shanghai Ninth People’s Hospital, Shanghai Jiao Tong University School of Medicine, Shanghai, China; 2 Department of Pathophysiology, Key Laboratory of Cell Differentiation and Apoptosis of National Ministry of Education, Shanghai Frontiers Science Center of Cellular Homeostasis and Human Diseases, Shanghai Jiao Tong University School of Medicine, Shanghai, China; 3 Laboratory for Reproductive Immunology, Hospital of Obstetrics and Gynecology, Shanghai Medical School, Fudan University, Shanghai, China

**Keywords:** Cell growth, Infertility, Phosphoinositol signalling

## Abstract

Endometriosis, an estrogen-dependent chronic inflammatory condition, afflicts reproductive-aged women. However, the underlying pathological mechanisms remain to be elucidated. Nicotinamide N-methyltransferase (NNMT) is a critical enzyme involved in cellular metabolism and methylation regulation. This study investigated the role of NNMT in endometriosis. By analyzing datasets GSE5108, GSE7305, GSE141549, GSE23339, and GSE25628, we identified a significant overexpression of NNMT in the eutopic endometrium and ectopic lesions of endometriosis patients compared to normal endometrium. Furthermore, NNMT was upregulated in collected endometrioma specimens and isolated primary endometrial stromal cells (ESCs) compared to their respective controls. Inhibition of NNMT using JBSNF-000088 attenuated the proliferation, migration, and invasion of ESCs. In vivo, treatment of mouse models of endometriosis with JBSNF-000088 resulted in a marked reduction in lesion weight and quantity. NNMT expression in ESCs was dose-dependently upregulated by 17β-estradiol at concentrations of 1 nM, 10 nM, and 100 nM, an effect that was attenuated by 10 nM progesterone. Additionally, treating HESCs with macrophage-conditioned medium elevated NNMT expression at both mRNA and protein levels. Knockdown of NNMT impeded the proliferation, migration, and invasion of ESCs, which was paralleled by decreased phosphorylation levels of Erb-b2 receptor tyrosine kinase 4 (ERBB4), PI3K, and AKT. Conversely, overexpressing ERBB4 mitigated the NNMT knockdown-induced decline in phosphorylated PI3K and AKT and rescued the proliferation of ESCs. Altogether, these results indicate that the overexpression of NNMT induced by estrogen and macrophage interaction modulates ESC proliferation via the NNMT-ERBB4-PI3K/AKT signaling pathway, as well as promotes cellular migration and invasion, contributing to the development of endometriosis.

## Introduction

Endometriosis is a prevalent gynecological disorder characterized by the ectopic growth of endometrial tissue outside the uterine cavity, leading to chronic pelvic pain and infertility. As an estrogen-dependent chronic inflammatory disease, it affects 5–10% of women in their reproductive years, frequently resulting in a significant decline in quality of life [1]. The etiology and pathology of endometriosis remain to be fully elucidated. Challenges persist, including widespread diagnostic delays and unsatisfactory treatment outcomes, primarily due to the lack of effective biomarkers and targeted treatments [2]. Mounting evidence suggests that immune and metabolic dysfunctions may contribute to the development of endometriosis [3, 4]. Uncovering the molecular mechanisms involved could greatly enhance our understanding and management of the disease.

Macrophages play a crucial role in the development of chronic pelvic inflammation, as they are recruited to and undergo alternative activation in both endometriotic lesions and the endometrium [5]. The ectopic endometrial lesions and eutopic endometrium in patients with endometriosis exhibit immune profiles distinct from those of normal endometrium [6]. Furthermore, the functional alteration of endometrial stromal cells (ESCs) contributes to disease progression and endometriosis-associated infertility [7]. Cellular interactions, notably between ESCs and other cell types, might induce functional changes influencing disease progression [8–10]. Nevertheless, the understanding of the crosstalk between macrophages and ESCs and their implications for endometriosis remains limited.

Nicotinamide N‑methyltransferase (NNMT) is a methyltransferase that catalyzes the transfer of methyl from S-adenosylmethionine (SAM) to nicotinamide (NAM) and produces S-adenosylhomocysteine (SAH) and 1-methyl nicotinamide (MNAM). This reaction is central to nicotinamide adenine dinucleotide (NAD^+^) metabolism and methylation balance [11]. NNMT is abundantly expressed in the liver and moderate levels are found in the normal endometrium and ovaries. The pathogenic or protective roles of NNMT in diverse biological contexts are still being investigated [12]. Aberrant expression of NNMT has been linked to cellular metastasis [13], epithelial-mesenchymal transition [14] tumorigenesis [15] and prognosis [16]. A previous study reported an increase in NNMT expression in endometrial stromal cells following exposure to macrophage-conditioned medium [17]. However, the specific role of NNMT in endometriosis has not been clearly identified.

Erb-B2 receptor tyrosine kinase 4 (ERBB4), a member of the epidermal growth factor receptor family of receptor tyrosine kinases, has several tyrosine phosphorylation sites within its cytoplasmic domain, enabling diverse downstream signaling cascades. It may act as either a tumor suppressor or a driver depending on the context [18]. To date, few studies have addressed the role of ERBB4 in endometriosis. Additionally, the phosphoinositide 3-kinase (PI3K)/AKT signaling pathway is implicated in the regulation of complex cellular functions. The hyperactivity of this pathway in endometriosis has been linked to increased progesterone resistance [19], as well as enhanced cellular proliferation, invasion [20] and migration [21]. Nonetheless, the precise mechanisms triggering this abnormal activation warrant further investigation.

This study sought to investigate the role of NNMT in the progression of endometriosis. We assessed the expression of NNMT in ectopic lesions and explored its impact on the proliferation, migration, and invasion of ESCs both in vitro and in vivo. We also examined the potential factors modulating the expression of NNMT in ESCs, including hormone treatment and macrophage co-culture. Moreover, we revealed that NNMT affects cell proliferation via the ERBB4/PI3K/AKT signaling pathway. These findings could offer new insights into the pathogenesis of endometriosis and illuminate potential therapeutic strategies.

## Results

### NNMT is overexpressed in eutopic and ectopic endometrial tissues of patients with endometriosis

**Fig. 1:**
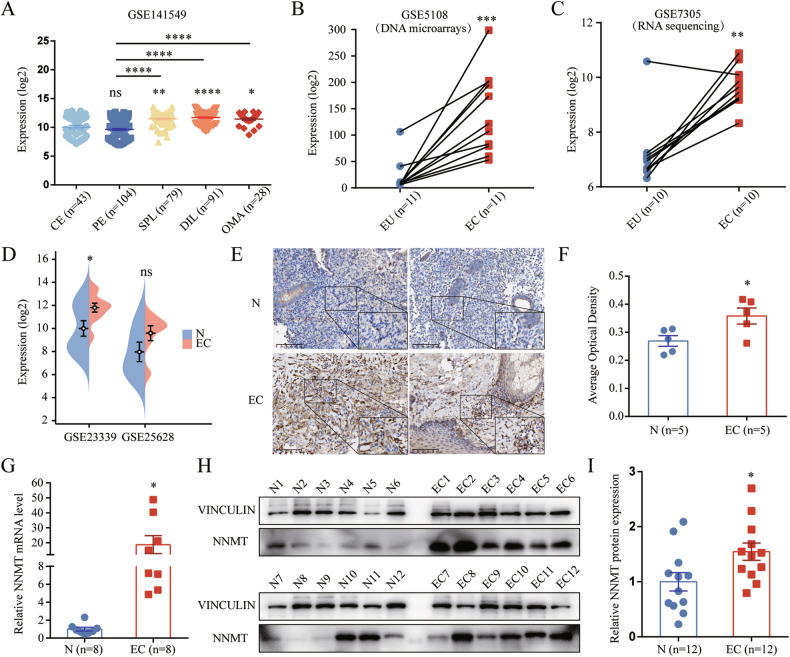
NNMT overexpression in eutopic and ectopic endometrial tissues of patients with endometriosis. **A** Comparative analysis of NNMT expression in control endometrium (CE, *n* = 43), eutopic patient endometrium (PE, *n* = 104), superficial peritoneal lesions (SPL, *n* = 79), deep infiltrating lesions (DIL, *n* = 91), and ovarian endometrioma (OMA, *n* = 28) utilizing dataset GSE141549. **B** NNMT expression in paired eutopic endometrium (EU, *n* = 11) and ectopic lesions (EC, *n* = 11) from dataset GSE5108. **C** NNMT expression in paired EU (*n* = 10) and EC (*n* = 10) from dataset GSE7305. **D** Comparison of NNMT expression in normal endometrium (N) and EC from datasets GSE23339 (N, *n* = 9; EC, *n* = 10) and GSE25628 (N, *n* = 6; EC, *n* = 7). **E** Representative immunohistochemistry staining images showing NNMT distribution in N and EC tissues. **F** Quantification of NNMT staining intensity in N (*n* = 5) and EC (*n* = 5) via ImageJ, presented as average optical density values. **G** NNMT mRNA levels in N (*n* = 8) and EC (*n* = 8) as determined by qRT-PCR, with β-ACTIN as the reference gene. **H** Western blot analysis of NNMT protein expression in N and EC, with VINCULIN serving as a loading control. **I** Quantification of NNMT protein levels in N (*n* = 12) and EC (*n* = 12) relative to VINCULIN. ns, not significant. *p < 0.05. **p < 0.01. ***p < 0.001. ****p < 0.0001.

To elucidate the role of NNMT in endometriosis, we initially explored its expression in endometrium and various types of endometriotic tissue using the Turku Endometriosis Database (GSE141549). Our analysis revealed higher NNMT expression levels in ectopic tissue from superficial peritoneal lesions, deep infiltrating lesions and ovarian endometrioma than in eutopic and control endometrium samples (Figs. S1, 1A). Additional analyses of published GEO datasets further supported that NNMT is significantly overexpressed in ectopic tissues relative to paired eutopic endometrium, as evidenced by dataset GSE5108 and dataset GSE7305 (Fig. 1B, C). Moreover, NNMT expression was elevated in ectopic tissue compared to the control endometrium in datasets GSE23339 and GSE25628 (Fig. 1D). To validate these observations, we collected ectopic lesion and normal endometrium specimens. Immunohistochemistry staining confirmed higher NNMT expression in ectopic tissues versus control endometrium (Fig. 1E, F), and elevated mRNA (Fig. 1G) and protein levels (Fig. 1H, I) were also detected in ectopic tissues. These results suggest a potential association between upregulated NNMT expression and endometriosis.

### Upregulated NNMT enhances the proliferation, migration and invasion capacities of ESCs

**Fig. 2:**
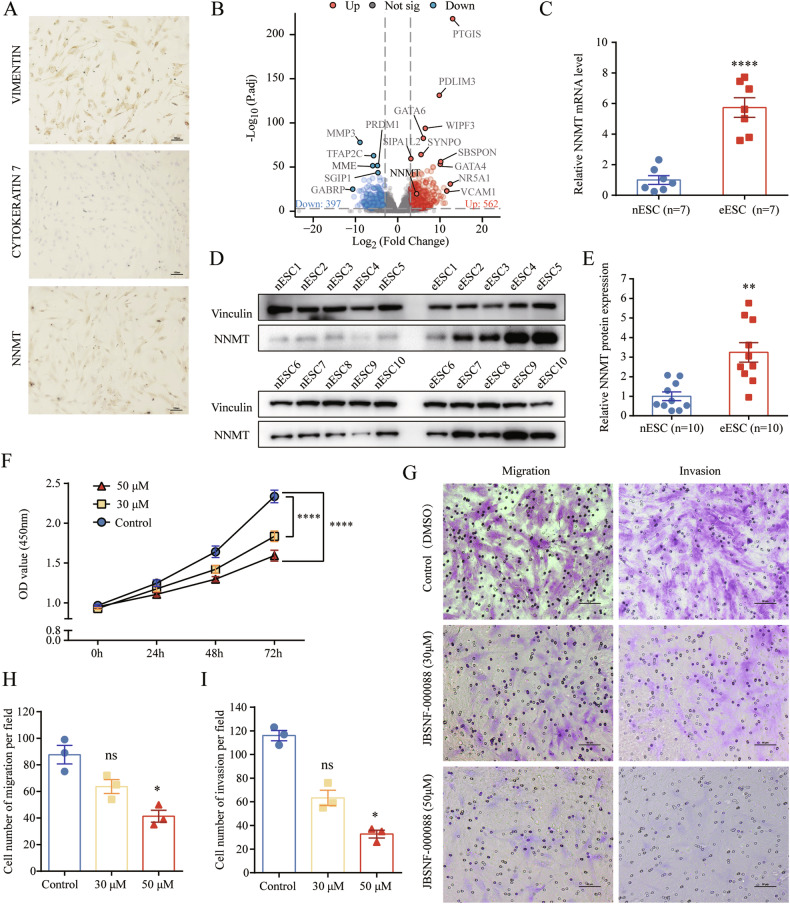
Upregulated NNMT facilitated the proliferation, migration and invasion capacity in ESCs. **A** Immunocytochemical characterization of primary endometrial stromal cells (ESC) using VIMENTIN and CYTOKERATIN 7 and demonstration of NNMT expression in ESCs. **B** Volcano plot displaying differential gene expression between normal endometrial stromal cells (nESC) and ectopic endometrial stromal cells (eESC) with a fold change >3 and false discovery rate <0.001 and NNMT was highlighted. **C** NNMT mRNA expression in nESC (*n* = 7) and eESC (*n* = 7) assessed by qRT-PCR with β-ACTIN as the reference gene. **D** Western blot analysis determining NNMT protein levels in nESC and eESC, with VINCULIN as the loading control. **E** Protein levels of NNMT in nESC (*n* = 10) and eESC (*n* = 10) relative to VINCULIN. **F** Impact of NNMT inhibitor JBSNF-000088 at 30 μM and 50 μM on eESC proliferation detected by CCK-8 assay. Data are presented as the mean ± SEM and analyzed using two-way ANOVA with Tukey’s post hoc test. **G** Effect of NNMT inhibitor JBSNF-000088 at 30 μM and 50 μM on migration and invasion of eESCs, evaluated by transwell assay. **H** Quantification of eESC migration per field (*n* = 3). **I** Quantification of eESC invasion per field (*n* = 3). The data in (**H**) and (**I**) were analyzed by one-way ANOVA. ns, not significant. *p < 0.05. **p < 0.01. ****p < 0.0001.

Given the extensive distribution of NNMT in the endometrial stroma observed via immunohistochemistry, we sought to define the expression and function of NNMT in ESCs. We initially isolated and identified primary ESCs (Fig. 2A). We then performed transcriptome sequencing of ectopic ESCs (eESCs) derived from endometriomas and normal ESCs (nESCs) from normal endometrium. NNMT was identified as one of the upregulated DEGs in eESCs in comparison to nESCs (Figs. S2, 2B). Subsequent analysis showed significant upregulation of NNMT mRNA (Fig. 2C) and protein (Fig. 2D, E) in eESCs relative to nESCs. To elucidate the role of NNMT in ESCs, we treated the eESCs with JBSNF-000088, an NNMT inhibitor, and assessed its impact on cellular functions. Treated eESCs exhibited reduced proliferation (Fig. 2F) and diminished migration and invasion capacities (Fig. 2G–I), suggesting a regulatory role for NNMT in cell proliferation, migration and invasion.

### Inhibition of NNMT suppresses endometriosis progression in vivo

**Fig. 3:**
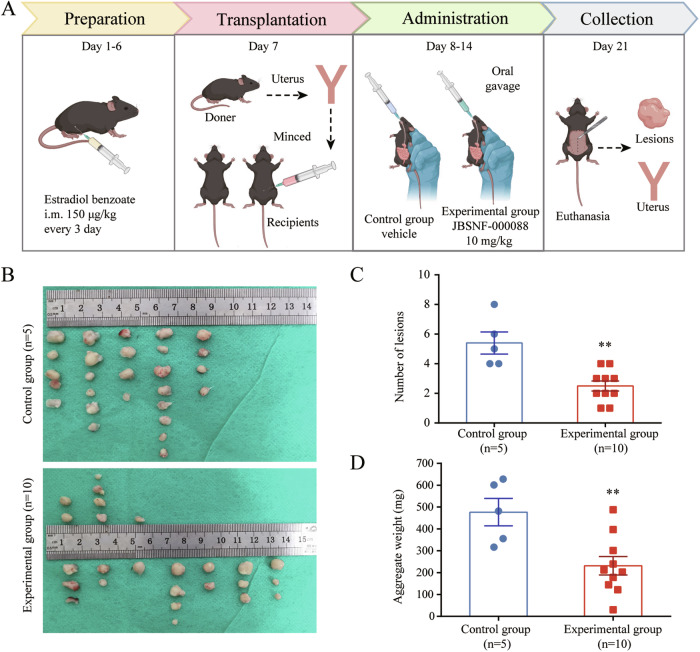
NNMT inhibition reduces endometriosis progression in vivo. **A** Schematic of the JBSNF-000088 treatment regimen in endometriosis mice models. **B** Representative ectopic lesions harvested on day 21 from control and JBSNF-000088-treated mice, with each column representing an individual mouse. **C** Lesion count comparison in endometriosis mice between control (*n* = 5) and JBSNF-000088 treatment groups (*n* = 10). **D** Comparison of the total lesion weight per mouse in control (*n* = 5) versus JBSNF-000088-treated mice (*n* = 10). **p < 0.01.

To study the effects of NNMT inhibition in vivo, we established the homologous transplanted models via intraperitoneal injection of endometrial tissue fragments. The experimental group received the NNMT inhibitor JBSNF-000088 intragastrically for seven consecutive days post-transplantation, whereas the control group was treated with the vehicle (Fig. 3A). On day 21, the resulting xenografts were harvested, enumerated and weighed (Fig. 3B). The experimental group showed a reduction in lesion number and weight compared to the control group (Fig. 3C, D), indicating that JBSNF-000088 treatment may attenuate endometriosis progression.

### Effects of 17β-estradiol treatment and macrophage co-culture on NNMT expression in HESCs

**Fig. 4:**
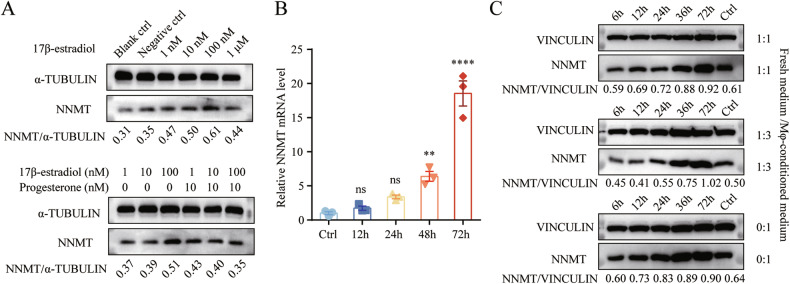
17β-estradiol treatment and macrophage co-culture upregulate NNMT expression in HESCs. **A** Western blot analysis of the impact of various concentrations of 17β-estradiol and progesterone on NNMT protein levels in HESCs, with α-TUBULIN serving as a loading control. **B** qRT-PCR analysis of the effect of macrophage-conditioned medium on NNMT mRNA levels in HESCs (*n* = 3), using β-ACTIN as the reference gene. The data were analyzed by one-way ANOVA. **C** Western blot determination of the effect of macrophage-conditioned medium on NNMT protein expression in HESCs, with VINCULIN as the loading control. ns, not significant. **p < 0.01. ****p < 0.0001.

Investigating the mechanisms underlying NNMT upregulation in eESCs, we considered the hormonal context of endometriosis. We examined the impact of 17β-estradiol and progesterone on NNMT gene expression. Treating HESCs with 1 nM, 10 nM and 100 nM of 17β-estradiol resulted in a dose-dependent increase in NNMT protein expression compared to the untreated control, which fostered cell proliferation, migration, and invasion (Figs. S3A, S4A). Interestingly, the increasing trend was counteracted by the administration of 10 nM progesterone (Fig. 4A). Additionally, we explored the effect of macrophage and HESC interaction on NNMT expression levels. The macrophage-conditioned medium significantly upregulated NNMT, both at the mRNA (Fig. 4B) and protein levels (Fig. 4C) in HESCs. Moreover, macrophage conditioned medium increased the proliferation, migration, and invasion ability of endometrial stromal cells (Figs. S3B, S4B).

### Knockdown of NNMT restrains the proliferation, migration, and invasion of HESCs

**Fig. 5:**
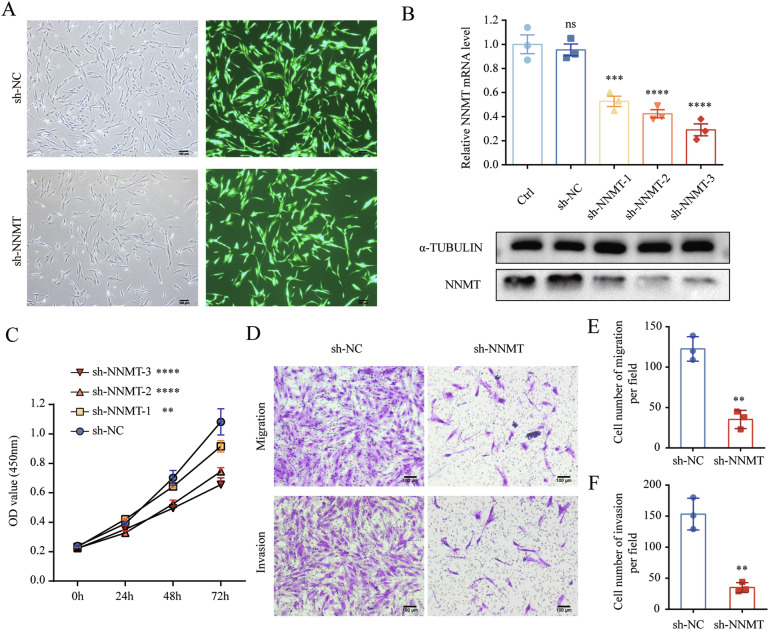
Knockdown of NNMT restrains the proliferation, migration and invasion of HESCs. **A** Fluorescence microscopy images of HESCs transfected with control and sh-NNMT lentiviruses. **B** Evaluation of sh-NNMT knockdown efficiency on NNMT mRNA (*n* = 3) and protein levels in HESCs by qRT-PCR with GAPDH as the reference gene and Western blot with α-TUBULIN as the loading control, respectively. The data were analyzed by one-way ANOVA. **C** CCK-8 assay detection of the impact of NNMT knockdown by three shRNAs on the proliferative capacity of HESCs (*n* = 3). Data are presented as the mean ± SEM and analyzed using two-way ANOVA. **D** Assessment of the influence of NNMT knockdown on the migration and invasion capacities of HESCs using transwell assay. **E** Quantification of migrated sh-NC-HESCs and sh-NNMT-HESCs per field (*n* = 3). **F** Quantification of invaded sh-NC-HESCs and sh-NNMT-HESCs per field (*n* = 3). ns, not significant. **p < 0.01. ***p < 0.001. ****p < 0.0001.

To further characterize the role of NNMT in endometriosis, HESCs were transfected with shRNA lentivirus targeting NNMT (Fig. 5A), and the efficiency of knockdown was analyzed (Fig. 5B). We observed significantly attenuated proliferation in sh-NNMT-HESCs compared to sh-NC-HESCs (Fig. 5C). Furthermore, the NNMT knockdown in HESCs significantly reduced migration and invasion capacities relative to the scramble control group (Fig. 5D–F), with an increase of NAD^+^ and SAM (Fig. S5).

### Downregulated NNMT impeded cell proliferation by inhibiting the ERBB4/PI3K/AKT signaling pathway in HESCs

**Fig. 6:**
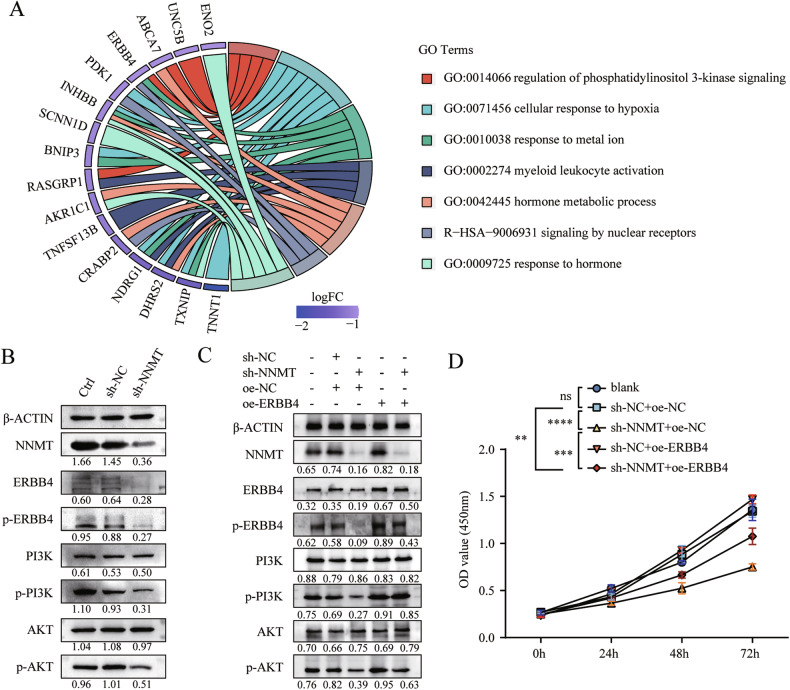
Downregulated NNMT hampers cell proliferation via the ERBB4/PI3K/AKT signaling pathway in HESCs. **A** Differentially expressed genes downregulated in sh-NNMT-HESCs compared to sh-NC-HESCs and their enriched terms. **B** Western blot analysis comparing the protein expression of ERBB4, Y1056-phosphorylated ERBB4 (p-ERBB4), PI3K, phosphorylated PI3K (p-PI3K), AKT, phosphorylated AKT (p-AKT) in sh-NNMT-HESCs to sh-NC-HESCs and the blank control, with β-ACTIN as the loading control. **C** Impact of ERBB4 overexpression on the protein expression of ERBB4, p-ERBB4, PI3K, p-PI3K, AKT, p-AKT in sh-NNMT-HESCs and sh-NC-HESCs. ERBB4, erb-b2 receptor tyrosine kinase 4. PI3K, phosphatidylinositol 3-kinase. **D** CCK-8 assay evaluating the effect of ERBB4 overexpression on the proliferation capacity of sh-NNMT-HESCs (*n* = 3). Data are presented as the mean ± SEM and analyzed using two-way ANOVA with Tukey’s post hoc test. ns, not significant. **p < 0.01. ****p < 0.0001.

We next sought to elucidate the potential functional mechanism of NNMT by conducting RNA sequencing and DEG analysis (Fig. S6). Enrichment analysis of these DEGs identified a downregulated PI3K/AKT signaling pathway with the highest enrichment score (Fig. 6A). Within this pathway, ERBB4, a member of the epidermal growth factor receptor family of receptor tyrosine kinases, was among the downregulated DEGs in sh-NNMT-HESCs compared with control cells (Fig. 6A). Moreover, previous studies have indicated that ERBB4 activation mediated diverse biological responses, including the regulation of cell growth, proliferation and migration. We subsequently confirmed the decreased expression of ERBB4 and the key components of the PI3K/AKT pathway. The results showed a reduction in ERBB4 protein levels and phosphorylation of Y1056 in sh-NNMT-HESCs (Fig. 6B). Additionally, NNMT knockdown significantly attenuated the phosphorylation of PI3K (p-PI3K) and AKT (p-AKT) (Fig. 6B). However, overexpression of ERBB4 upregulated p-PI3K and p-AKT levels, counteracting the decreases seen in sh-NNMT-HESCs (Fig. 6C). Rescue of the cell proliferation defects associated with NNMT knockdown was achieved upon ERBB4 overexpression (Fig. 6D). These findings suggest that NNMT stimulates cell proliferation through the ERBB4/PI3K/AKT axis. Furthermore, the results were verified by LC-MS /PRM analysis (Fig. S7).

## Discussion

In this study, we found that aberrantly high expression of NNMT is associated with the proliferation, migration and invasion of ESCs, contributing to the progression of endometriosis. As a metabolic enzyme, NNMT catalyzes the transfer of a methyl group from SAM to NAM, intimately linking methionine with NAD^+^ metabolism. This transfer, responsible for SAM consumption, affects gene expression and cellular function by altering histone methylation levels since SAM serves as the universal methyl donor for proteins, DNA, RNA, lipids and other cellular metabolites. NAM, a vitamin B3 derivative, precedes the formation of NAD^+^, an essential cofactor in numerous redox reactions and a modulator of signaling and transcriptional events [22]. Consequently, NNMT stands as a pivotal conduit between metabolism and epigenetic control, with its metabolites exerting tissue-specific influences on intracellular processes [23]. Elevations in NNMT and its metabolite MNAM are known to stabilize SIRT1, a critical regulator of gluconeogenesis, cholesterol synthesis in the liver, and defenses against diet-induced obesity [24]. Paradoxically, NNMT activity is associated with cell migration, invasion, and chemoresistance in cancer cells [25]. Knockdown of NNMT results in methyl overflow, which increased DNA and histone methylation, impairing metastatic colonization [26]. Positive correlations have been established between NNMT levels and the progression and prognosis of ovarian tumors [27, 28]. Consistent with these findings, our study implicates NNMT upregulation in ectopic lesions and the enhanced proliferation, migration and invasion of ESCs. Prior studies have indicated NNMT’s role in AKT activation during oncogenesis [15], cell morphology alteration [29], and the development of drug resistance [30]. Our results echo these findings, as downregulated NNMT led to PI3K/AKT signaling pathway inactivation and subdued the proliferation of ESCs. Further research has been dedicated to exploring NNMT-related metabolic and epigenetic alteration in endometriosis.

Considering the elevated levels of NNMT and local estrogen augmentation in endometriotic tissue [31], we investigated the potential of estrogen to drive NNMT upregulation. Treatment with 17β-estradiol increased NNMT protein levels in HESCs, with dose-dependent elevation observed between 1 nM and 100 nM. Beyond this threshold, additional increases in 17β-estradiol did not further NNMT protein expression. Several potential biological mechanisms might explain the observed plateau at the higher concentration of 17β-estradiol, including receptor and pathway saturation [32, 33]; non-linear dose-responses [34]; epigenetic modulation [35]; differential signaling pathway activation; regulatory feedback mechanisms; and cytotoxic effects at high 17β-estradiol doses. Importantly, progesterone treatment attenuated the 17β-estradiol-mediated increase of NNMT. These observations suggest that NNMT expression may be tightly linked to cyclical hormonal changes. A panel including NNMT has previously distinguished between mid-secretory and early secretory endometrial samples and differentiated control from recurrent implantation failure mid-secretory endometrium [36]. Furthermore, NNMT-stabilized SIRT1 plays a role in decidualization defects [37], progesterone resistance and progression of endometriosis [38]. Additionally, as one of the few genes uniformly expressed during the window of implantation, NNMT is likely involved in the mechanisms of embryo implantation [39]. The sensitivity of NNMT expression to hormones dynamics, and its possible effects on decidualization and implantation should be investigated in future studies.

Our results also show that macrophage-conditioned media augmented NNMT expression in ESCs. There is a growing body of evidence indicating that macrophages are instrumental in developing ectopic lesions and associated inflammation due to their abundance and phenotypic versatility [40]. The emerging significance of intercellular communication between macrophages and ESCs in endometriosis has been receiving increasing focus [41]. Macrophages impact ESC autophagy, proliferation, and migration through the secretion of extracellular vesicles [42, 43] and cytokines [44] via ligand-receptor interactions [45]. A recent study revealed that NNMT could promote M2 polarization of macrophages in gallbladder carcinoma [46]. The exact mechanisms through which macrophages induce NNMT expression in ESCs and the role of NNMT in endometriosis-related macrophage function are compelling subjects for future investigation.

After NNMT knockdown, we observed a reduction in ERBB4 protein level and its phosphorylation in HESCs. ERBB4 is a well-characterized receptor that acts as both a tumor suppressor and an oncoprotein in different contexts. Its phosphorylation generates various binding sites that facilitate diverse cytoplasmic signaling cascades [18]. The expression and stability of ERBB4 can be regulated by somatic mutation [47], epigenetic modification [48], ligands [49], and specific non-coding RNA [50, 51]. Phosphorylation at the Y1056 residue within the YXXM consensus motif of ERBB4 leads to the recruitment of the p85 adaptor unit and subsequent activation of the PI3K/AKT signaling pathway [52]. AKT is involved in the regulation of cell apoptosis and proliferation, and increased AKT activity has been implicated in the establishment of ectopic endometrial tissues [53]. Our study demonstrated that overexpression of ERBB4 upregulated p-PI3K and p-AKT in ESCs. This overexpression rescued the impaired proliferation caused by NNMT knockdown, providing novel insights into the pathogenesis of endometriosis.

From a therapeutic perspective, NNMT has garnered increasing interest as a potential target [54]. JBSNF-00008 (6-Methoxynicotinamide) is a potent, orally active NNMT inhibitor. This compound has shown efficacy in insulin sensitization, glucose modulation, and body weight reduction in diet-induced obese mice models of diabetes, as well as in protecting against alcoholic fatty liver disease [55] and gallbladder carcinoma [46] in murine models. Our findings indicate that JBSNF-00008 significantly alleviated the progression of endometriosis in vivo, suggesting that NNMT is a promising therapeutic target. Detailed characterizations of NNMT could pave the way for the development of new inhibitors for treating endometriosis.

However, the study was limited by the small sample size and the monotypic nature of the samples. While bioinformatic evidence from multiple types was provided, our investigation was concentrated on endometrium and ovarian endometrioma samples from the proliferative phase due to accessibility constraints, which limits the generalizability of our findings. Accordingly, further research is necessary to elucidate the role of NNMT and the ERBB4/PI3K/AKT signaling pathway in the pathophysiology of endometriosis.

In summary, our work indicates that NNMT is abnormally overexpressed in endometrioma, as confirmed by bioinformatics analysis and validation in clinical specimens. Suppression of NNMT jeopardized the proliferation, migration, and invasion capabilities of ESCs in vitro and ameliorated the development of endometriosis in vivo. Diminished NNMT expression in ESCs attenuated the expression and phosphorylation of ERBB4, leading to the inactivation of the PI3K/AKT signaling pathway, which ultimately impaired cell proliferation. Moreover, our findings suggest that estrogen and macrophages may play roles in upregulating NNMT in ESCs. These insights extend our understanding of the pathophysiological mechanisms in endometriosis and could inform future therapeutic interventions.

## Materials and methods

### Patients and samples

This study was approved by the Ethics Committee of Shanghai Ninth People’s Hospital and performed in compliance with the Declaration of Helsinki. Written informed consent was obtained from all participants and confirmed by the Ethics Committee. We collected the cyst wall of endometrioma (*n* = 20) from patients with histologically confirmed endometriosis and normal endometrial tissues (*n* = 18) from laparoscopic-confirmed endometriosis-free patients who underwent simultaneous uterine curettage between July 2020 and December 2021. All participants had regular menstrual cycles and received surgery in their proliferative phase. Patients with pelvic infection, reproductive abnormalities, abnormal uterine bleeding, malignant tumors, and those who received any hormone therapy within six months before the collection were excluded. The samples were used to isolate primary cells or stored in liquid nitrogen for preservation.

### Primary endometrial stromal cell isolation and culture

Primary ESCs were isolated as previous descriptions with some modifications [56]. Briefly, the endometrial tissues were minced and digested with 0.1% type-IV collagenase (C5138-1G, Sigma-Aldrich, MO, USA) for 30–45 min at 37 °C on a shaker. Subsequently, the tissue fragments were filtered through a 40-μm cell strainer (#352340, Corning Falcon, NY, USA), and the resulting suspension was centrifuged at 1200 rpm for 5 min at room temperature to collect primary cells. The primary cells were used for experiments at passages 3 to 5. The Human Endometrial Stromal Cells (HESCs) were purchased from the American Type Culture Collection and authenticated using Short Tandem Repeat analysis. The primary endometrial stromal cells and HESCs were cultured in DMEM/F12 medium (SH30023.01b, HyClone, UT, USA) supplemented with 10% fetal bovine serum (FBS, #0500, ScienCell, CA, USA) and 1% penicillin/streptomycin/amphotericin B (C125C8, NCM Biotech, Suzhou, China) at 37 °C in a humidified atmosphere with 5% CO_2_. The culture medium was refreshed every other day. When needed, NNMT inhibitor JBSNF-000088 (HY-112584, MedChemExpress, NJ, USA), 17β-estradiol (E‐2758, Sigma-Aldrich, USA) and progesterone (P0130, Sigma-Aldrich, USA) were administered to the cells.

### Culture and polarization of THP-1 cells

THP-1 cells were cultured in RPMI-1640 medium (SH30809.01, HyClone, USA) supplemented with 10% FBS and 1% penicillin/streptomycin/amphotericin B. The cells in 6-well plates (5 × 10^5^/well) were treated with 100 ng/mL phorbol 12-myristate 13-acetate (PMA, HY-18739, MedChemExpress, USA) for 24 h to obtain adherent M0 macrophages. Subsequently, the media was replaced with PMA-free RPMI-1640 containing 10% FBS and 1% penicillin/streptomycin/amphotericin B. After 24 h, the supernatant was collected as the conditioned medium.

### Indirect co‐culture system

To establish an indirect co‐culture system between HESCs and macrophages, we used the 24-h-cultured supernatant of M0 macrophages to incubate HESCs. After 24 h of incubation, the HESCs were harvested for analysis.

### Cell transfection

For NNMT knockdown, three specifically designed shRNAs cloned into pGMLV-SC5-puro vectors and scrambled control vectors were transfected into 293T cells using the lentiviral packaging mix to produce viruses for 48 h. The supernatant containing the virus was filtered through a 0.45-μm filter and used to infect HESCs. Seventy-two hours later, the infected HESCs were selected with 30-μg/mL puromycin (A1113803, Invitrogen, CA, USA), and the knockdown efficiency was detected. For ERBB4 overexpression, the vectors were transfected into HESCs using Lipofectamine 3000 (L3000008, Invitrogen, USA) according to the manufacturer’s instructions. The sequences of shRNAs of NNMT were as follows:

**Table:** 

oligos	DNA sequence (5′ to 3′)
Primer-NC-T	GATCTGTTCTCCGAACGTGTCACGTTTCAAGAGAACGTGACACGTTCGGAGAATTTTTTC
Primer-NC-B	AATTGAAAAAATTCTCCGAACGTGTCACGTTCTCTTGAAACGTGACACGTTCGGAGAACA
Primer-T1	GATCCGCGCTCAAGAGCAGCTACTACCTCGAGGTAGTAGCTGCTCTTGAGCGCTTTTTT
Primer-B1	AATTAAAAAAGCGCTCAAGAGCAGCTACTACCTCGAGGTAGTAGCTGCTCTTGAGCGCG
Primer-T2	GATCCGCTGTGAAAGAGGCTGGCTACCTCGAGGTAGCCAGCCTCTTTCACAGCTTTTTT
Primer-B2	AATTAAAAAAGCTGTGAAAGAGGCTGGCTACCTCGAGGTAGCCAGCCTCTTTCACAGCG
Primer-T3	GATCCGGTGATCTCGCAAAGTTATTCCTCGAGGAATAACTTTGCGAGATCACCTTTTTT
Primer-B3	AATTAAAAAAGGTGATCTCGCAAAGTTATTCCTCGAGGAATAACTTTGCGAGATCACCG

### RNA extraction and quantitative real-time polymerase chain reaction (qRT-PCR)

Total RNA from tissues and cells was extracted with TRIzol Reagent (#9108, RNAiso plus, Taraka, Japan), and cDNA was obtained with a reverse transcription kit (#6110A, PrimeScript™ RT Master Mix, Takara, Japan) following the manufacturer’s instructions. QRT-PCR was conducted using the SYBR Green-based kit (RR820A, TB Green Premix Ex Taq Mix, Takara, Japan). Gene expression levels were calculated using the 2 ^−ΔΔCT^ method with β-ACTIN as the endogenous reference gene. The primer sequence information is as follows:

- NNMT forward primer sequence: AAGATATTCTGCCTAGACGGTG
- NNMT reverse primer sequence: AGCAGAGAGGAGCTGATAGATA
- GAPDH forward primer sequence: CAACGTGTCAGTGGTGGACCTG
- GAPDH reverse primer sequence: GTGTCGCTGTTGAAGTCAGAGGA
- ACTB forward primer sequence: GGCACCACACCTTCTACAATGAGC
- ACTB reverse primer sequence: GATAGCACAGCCTGGATAGCAACG
- ERBB4 forward primer sequence: GTTCAGGATGTGGACGTTGC
- ERBB4 reverse primer sequence: CTGCCGTCACATTGTTCTGC

### Next-generation RNA Sequencing

Total RNA was isolated from primary eESCs, nESCs, sh-NNMT-HESCs, and sh-NC-HESCs. The RNA quantities and qualities were evaluated using a NanoDrop ND-8000 spectrophotometer and an Agilent Bioanalyzer. RNA sequencing was performed using the Illumina HiSeq system by NovelBio (Shanghai, China). The DESeq2 algorithm was used for screening to obtain the differential expressed genes (DEGs) with fold change >2 and false discovery rate <0.05. These data were deposited into the National Center for Biotechnology Information (NCBI) Sequence Read Archive (SRA) under accession number PRJNA1054992 and are also available in Figshare at the following URL: https://figshare.com/s/8f2b22ecda3aeda9efe8.

### Detection of NAD^+^ and SAM

NAD^+^ levels were detected using the WST-8 assay (S0175, Beyotime, China) and SAM levels were measured using the ELISA kit (JZT-61728H2, Jiaoziteng, China) according to the manufacturer’s instructions.

### Liquid chromatography (LC)-mass spectrometry (MS) and parallel reaction monitoring (PRM) analysis

The expression levels of selected proteins were quantified by LC-MS /PRM with technical support of Shanghai Bioprofile Technology. Peptides were prepared according to the iTRAQ/TMT/label-free protocol. PRM analysis was performed on a Q Exactive Plus mass spectrometer (Thermo Scientific). The mass spectrometer was operated in positive ion mode and with the following parameters: The full MS1 scan was acquired with a resolution of 70,000 (at 200 m/z), automatic gain control (AGC) target values of 3.0 × 10^6^, and a maximum ion injection time of 250 ms. Full MS scans were followed by 20 PRM scans at 35,000 resolution (at m/z 200) with AGC 3.0 × 10^6^ and a maximum injection time 200 ms. The targeted peptides were isolated with a 2Th window and fragmented at normalized collision energy of 27 in a higher energy dissociation (HCD) collision cell. The raw data was analyzed using Skyline (MacCoss Lab, University of Washington) to obtain the signal intensities of individual peptide sequences.

### Western blot

The cells and tissue pieces were washed with precooled phosphate-buffered saline and treated with RIPA buffer (P0013C, Beyotime, Jiangsu, China) for 30 min on ice. The lysate was collected and centrifuged at 4 °C at 12,000 rpm for 20 min. The supernatant was used to determine protein concentration using the BCA method (P0010, Beyotime, China). The remaining supernatant was heated at 95 °C for 10 min with loading buffer (P0015L, Beyotime, China). Proteins were then separated by SDS-PAGE electrophoresis and transferred to polyvinylidene fluoride membranes (IPVH00010, Millipore, MA, USA). The membranes were blocked with 5% skim milk or bovine serum albumin for one hour at room temperature and then incubated with primary rabbit antibodies against NNMT (ab119758, Abcam, Cambridge, UK), VINCULIN (#13901, Cell Signaling Technology, MA, USA), α-TUBULIN (ab52866, Abcam, UK), β-ACTIN (AF7018, Affinity Biosciences, Jiangsu, China), ERBB4 (AF6445, Affinity Biosciences, USA), phospho-ERBB4 (AF8370, Affinity Biosciences, USA), PI3K (A23303, ABclonal, Wuhan, China), phospho-PI3K (AP0854, ABclonal, China), AKT (#4691T, Cell Signaling Technology, USA), and phospho-AKT (#4058T, Cell Signaling Technology, USA) at 4 °C overnight. Membranes were then incubated with horseradish peroxidase-conjugated secondary antibodies for 1.5 h at room temperature. Protein bands were visualized using enhanced chemiluminescence (raw data in Supplementary File 2) and analyzed using Image J software (version 1.41).

### Cell proliferation assay

Cell proliferation was evaluated using the Cell Count Kit-8 (CK04, CCK-8, Dojindo, Japan) according to the manufacturer’s protocols. One thousand cells were seeded into each well of a 96-well plate and incubated for 24 h at 37 °C with 5% CO_2_. Then, 10 μL of CCK-8 solution was added to each well and incubated for 40 min in the dark at 37 °C. The absorbance of each well was measured at a wavelength of 450 nm at 0, 24, 48, 72, and 96 h using a spectrophotometer microplate reader.

### Transwell invasion and migration assay

For the cell invasion experiment, the transwell chamber was pre-treated with diluted Matrigel (YZ-354234, BD Bioscience, NJ, USA). Subsequently, 5 × 10^4^ cells in 200 μL of FBS-free medium were seeded into the upper chamber and 600 μL of complete medium was added to the lower chamber. After 24 and 48 h, non-invasive and non-migratory cells were removed, while the migratory and invasive cells were fixed with 4% paraformaldehyde and stained with crystal violet (C0121, Beyotime, China). The stained cells were observed, photographed and counted under an optical microscope.

### Immunohistochemistry (IHC) and immunocytochemistry (ICC)

For immunohistochemistry, paraffin-embedded uterine and ectopic tissues were sectioned. The sections were dewaxed, hydrated, subjected to antigen retrieval, and incubated with 3% H_2_O_2_ for 20 min to quench endogenous peroxidase activity. Then sections were blocked with 5% BSA and incubated with the primary antibodies against NNMT (ab119758, Abcam, UK), VIMENTIN (#5741T, Cell Signaling Technology, USA), and CYTOKERATIN 7 (ab68459, Abcam, UK) at 4 °C overnight, followed by incubation with horseradish peroxidase-conjugated secondary antibodies the next day. After staining with DAB chromogen (P0203, Beyotime, China), sections were briefly counterstained with hematoxylin. The staining intensity was analyzed using Image J software (version 1.41). The immunocytochemistry protocol was similar, with cells cultured on glass coverslips, fixed with 4% paraformaldehyde, and permeabilized with 0.3% Triton X-100 before following the same steps as for immunohistochemistry.

### Bioinformatics analysis

Gene expression in the endometrium, peritoneum and various endometriosis lesions from controls and endometriosis patients was analyzed using the publicly accessible Turku Endometriosis Database (https://endometdb.utu.fi/). Transcriptomic data profiles of datasets GSE141549, GSE7305, GSE5108, GSE23339 and GSE25628 were downloaded from the Gene Expression Omnibus database (https://www.ncbi.nlm.nih.gov/geo/). Data were analyzed with the R package stats (version 4.2.1) and car (version 3.1.0) and visualized with ggplot2 (version 3.3.6). Pathway enrichment analyses were conducted using the Metascape web tool (http://metascape.org), with Gene Ontology (GO) items considered significantly enriched at a *p*-value of <0.01, a minimum count of 3 and an enrichment factor of >1.5.

### Mouse model of endometriosis

The animal experiments were approved by the Ethics Committee of Shanghai Ninth People’s Hospital. All experiments were performed in accordance with the approved protocol and relevant guidelines and regulations. Six-week-old C57BL/6N female mice were purchased from Shanghai Jiesijie Experimental Animal Co., Ltd (Shanghai, China). The sample sizes were determined based on available resources and ethical considerations. The mice were housed in a specific pathogen-free environment under regulated 12-h light/dark cycles at 23–25 °C and acclimatized to the environment for two weeks before the experiment. Estradiol benzoate (150 μg/kg) was injected intramuscularly into all mice on day one and day four to synchronize their estrous cycles and promote endometrial growth. On day seven, minced uterine fragments from one donor mouse were intraperitoneally injected into two recipient mice as described previously [57]. Mice were randomized into control and experimental groups. From day nine to day thirteen, the experimental group received intragastric administration of JBSNF-000088 (10 mg/kg, HY-112584, MedChemExpress, USA) once daily, while the control group received an equal volume of vehicle. Mice were sacrificed on day twenty-one, and uterine and ectopic lesions were collected for measurement.

### Statistical analysis

Each experiment was performed independently with a minimum of three replicates for data collection. Unless otherwise specified, data were presented as means ± standard deviation (SD) and were analyzed using GraphPad Prism 7 software (GraphPad Software Inc., San Diego, CA, USA). For comparisons between two groups, an independent two-sample Student’s *t*-test was utilized. For comparisons involving more than two groups, a one-way analysis of variance (ANOVA) was performed. For analyses involving two independent variables, a two-way ANOVA was conducted. Following a significant ANOVA result, post-hoc pairwise comparisons were conducted using Tukey’s Honest Significant Difference (HSD) test to identify specific differences between groups. Statistical significance was determined at a *p*-value threshold of less than 0.05. All statistical tests were two-tailed.

## Supplementary information

Supplementary figures

Original western blots

## Data Availability

The original contributions presented in the study are included in the article and Supplementary Material. Supplementary information is available at the Cell Death Discovery’s website. Further inquiries can be directed to the corresponding author.
