# Supplementary material for: Overexpressed nicotinamide N‑methyltransferase in endometrial stromal cells induced by macrophages and estradiol contributes to cell proliferation in endometriosis
Source: Cell Death Discov. 2024 Nov 3;10:463. doi: 10.1038/s41420-024-02229-3 (PMC11532478; doi:10.1038/s41420-024-02229-3)
Supplement: Supplementary file 1 — Supplementary figures [file 41420_2024_2229_MOESM1_ESM.docx]

**Figure S1**

**S1A**


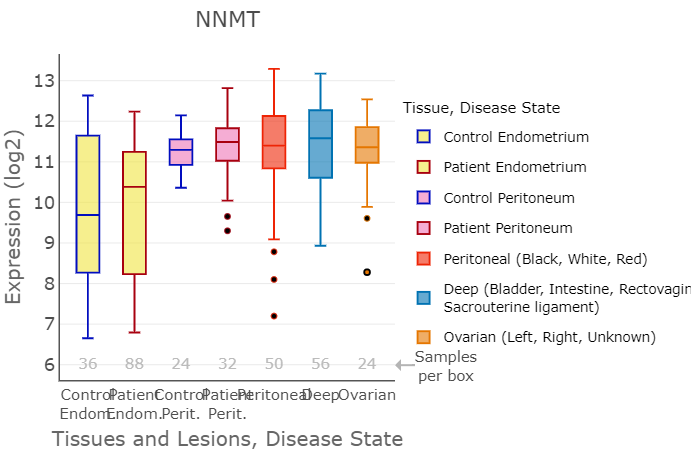


**S1B**


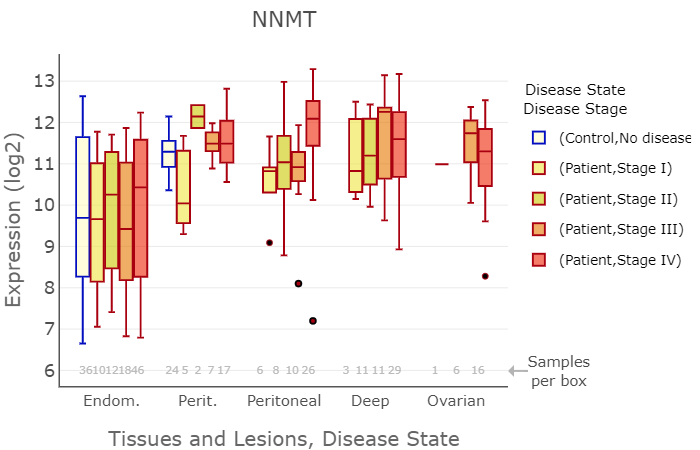


**Figure S1. NNMT tends to elevate in ectopic tissue and is related to the severity of EMS according to the Turku Endometriosis Database.** (A) The expression levels of NNMT in superficial peritoneal lesions, deep infiltrating lesions, and ovarian endometriomas showed an increasing trend compared to control endometrium and patient endometrium samples. (B) An upward trend of NNMT expression was observed in lesion tissues with increasing disease severity.

**Figure S2**


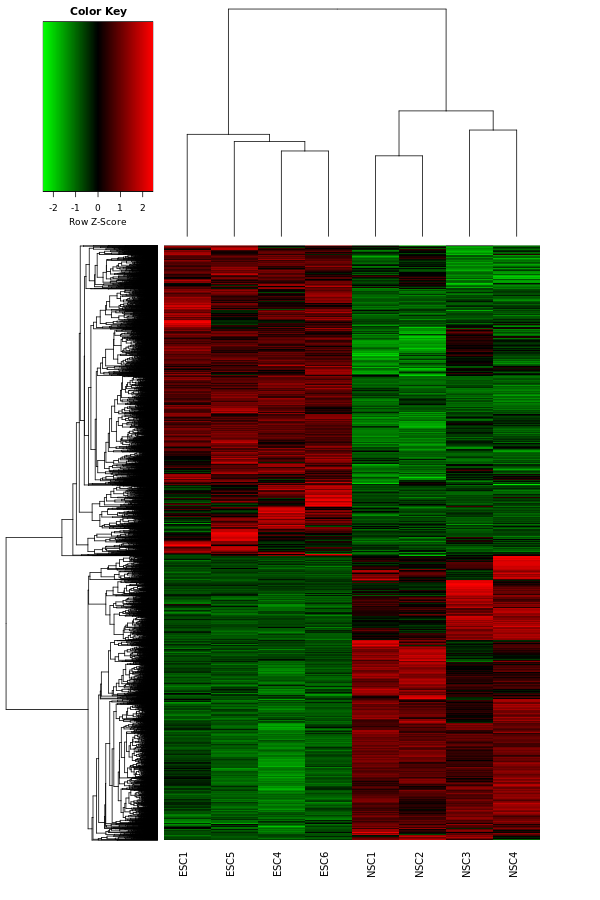


**Figure S2. The clustered heatmap of the differentially expressed genes between four normal endometrial stromal cells (nESC) and four ectopic endometrial stromal cells (eESC).**

**Figure S3**

**S3A**


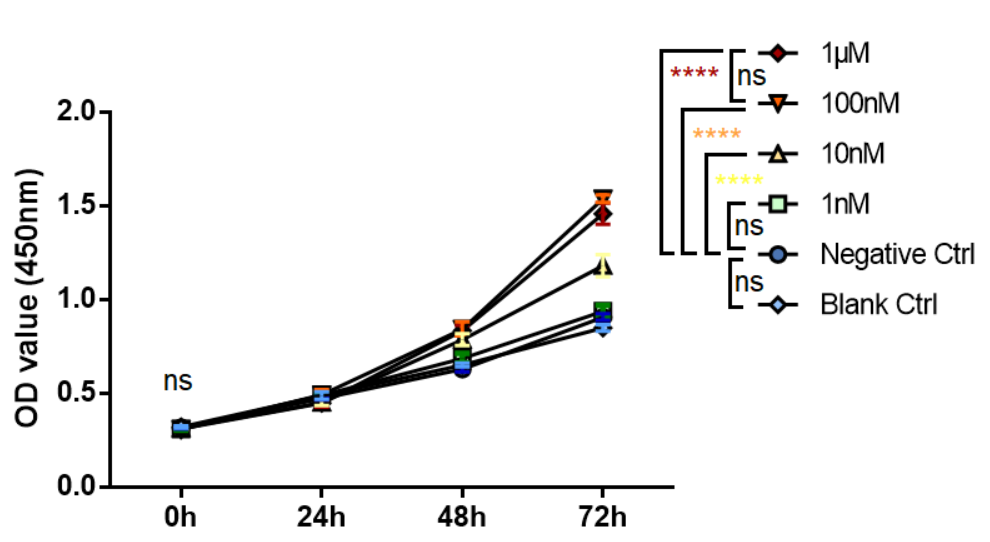


**S3B**


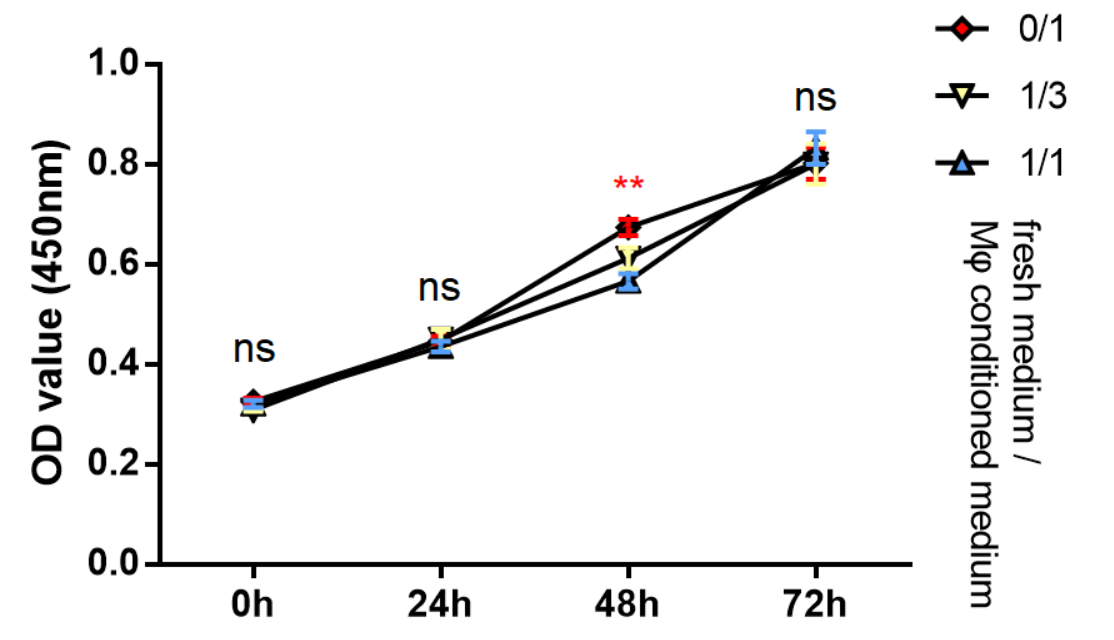


**Figure S3. Effects of 17β-estradiol treatment and macrophage co-culture on the proliferation capacity of HESCs.** (A) The effects of 17β-estradiol at 1 nM, 10 nM, 100 nM, and 1 μM on the proliferation capacity of HESCs were detected by CCK-8 assay. (B) The effects of macrophage-conditioned medium mixed with fresh medium in different proportions on the proliferation capacity of HESCs were detected by CCK-8 assay. Data are presented as the mean ± SEM and analyzed using two-way ANOVA. Tukey’s post hoc test was used to determine significance. ns, not significant. * p<0.05, ** p<0.01, *** p<0.001, **** p<0.0001.

**Figure S4**

**S4A**


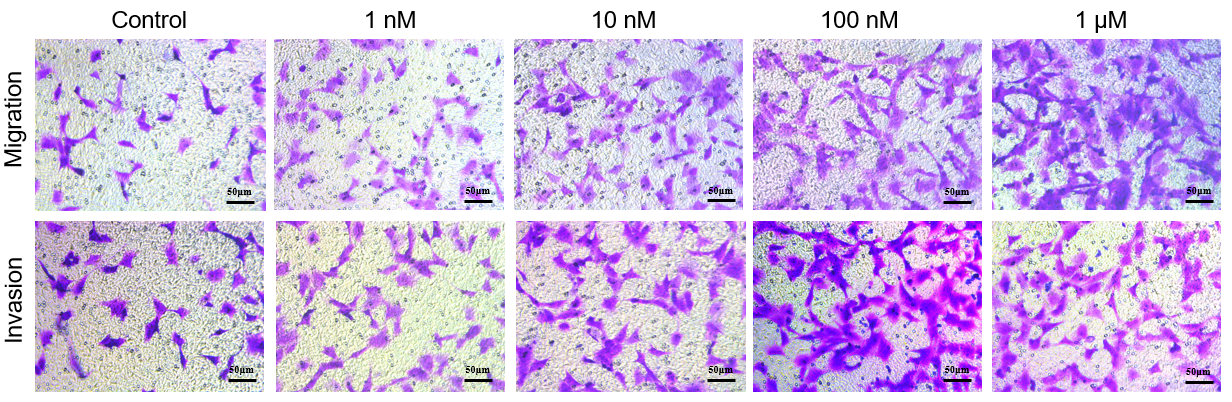


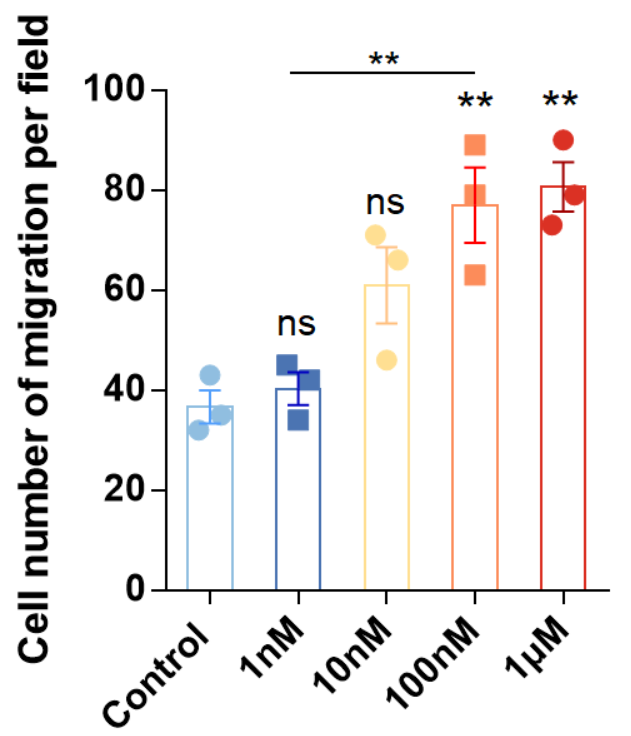

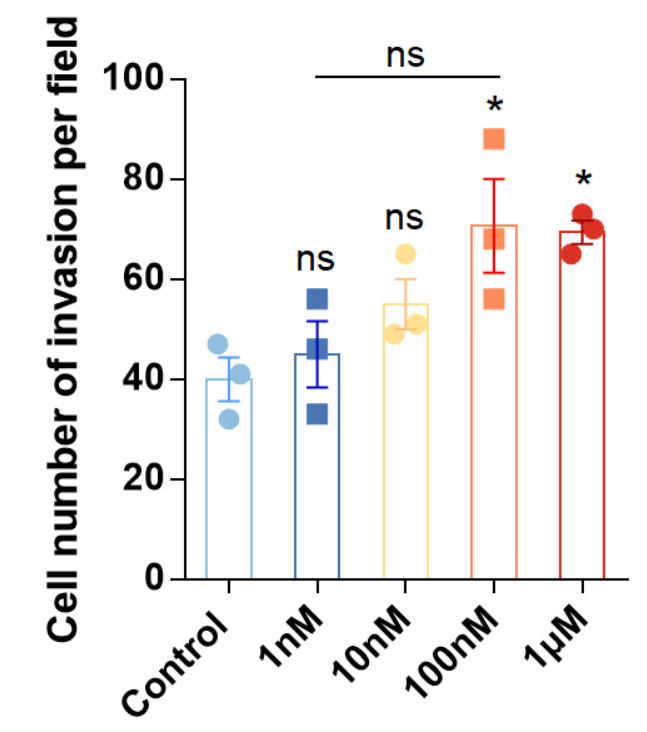


**S4B**


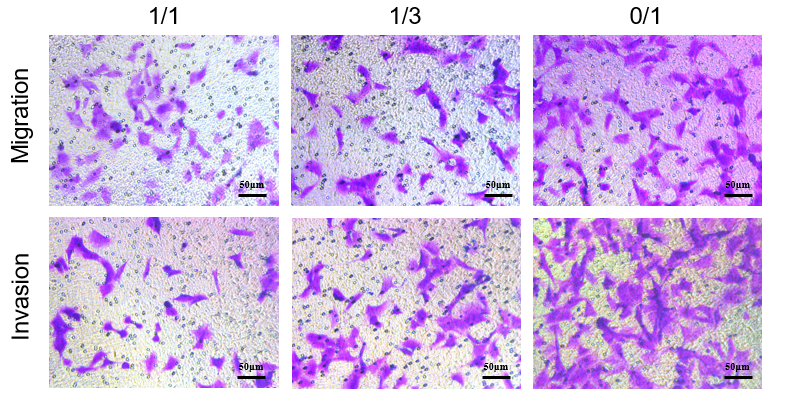


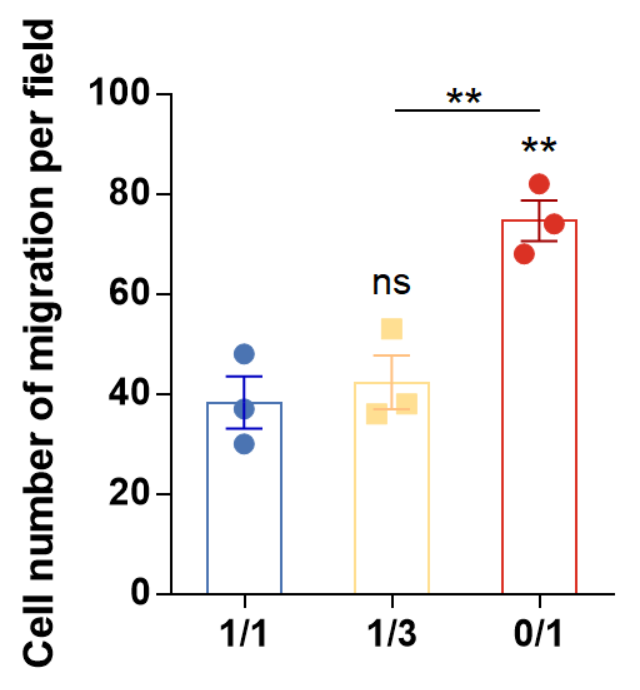

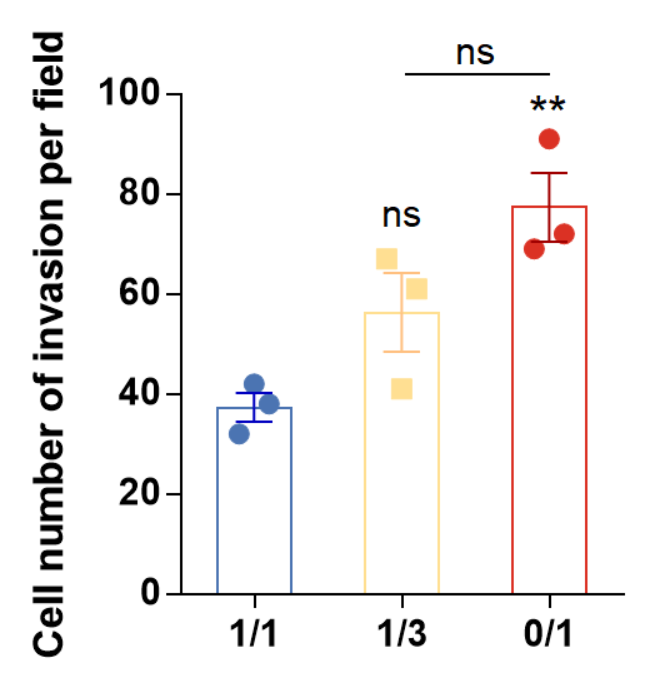


**Figure S4. Effects of 17β-estradiol treatment and macrophage co-culture on the migration and invasion capacity of HESCs.** (A) The effects of 17β-estradiol at 1 nM, 10 nM, 100 nM, and 1 μM on the migration and invasion capacity of HESCs were detected by transwell assay (n = 3). (B) The effects of macrophage-conditioned medium mixed with fresh medium in different proportions on the migration and invasion capacity of HESCs were detected by transwell assay (n = 3). The average number of invaded HESCs per field is presented as the mean ± SEM and analyzed by one-way ANOVA. ns, not significant. * p<0.05, ** p<0.01, *** p<0.001, **** p<0.0001.

**Figure S5**

**S5A**


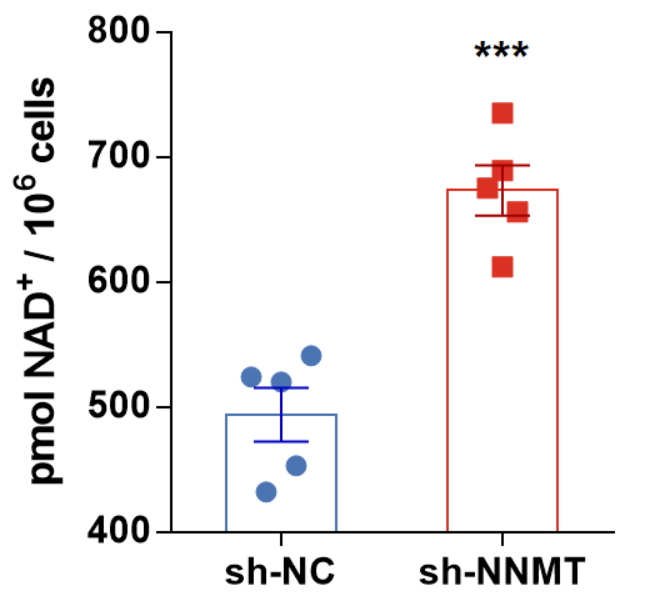


**S5B**


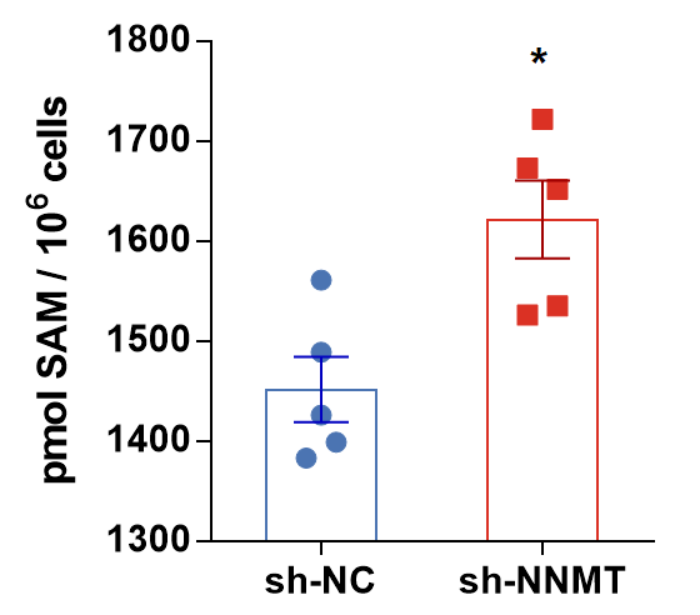


**Figure S5. Quantification of** **NAD⁺ and SAM after NNMT knockdown in HESCs.** (A) NAD⁺ levels were upregulated in sh-NNMT cells (n = 5). (B) SAM levels were upregulated in sh-NNMT cells (n = 5). Data are presented as the mean ± SEM and analyzed using Student’s t-test. ns, not significant. * p<0.05, ** p<0.01, *** p<0.001, **** p<0.0001.

**Figure S6**


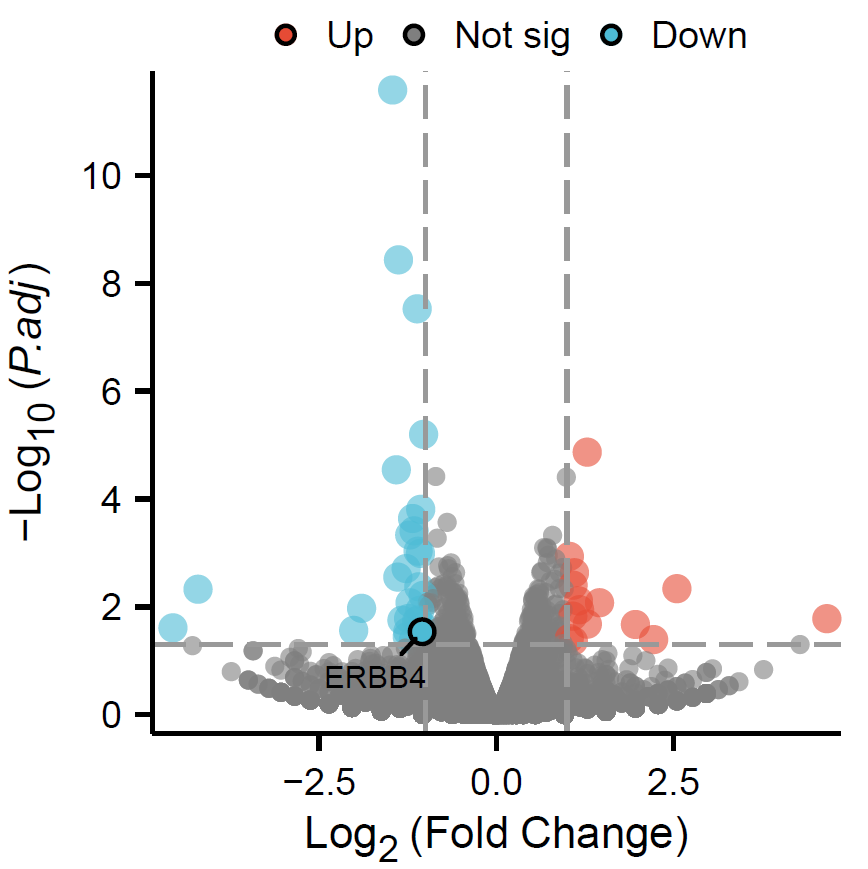


**Figure S6. The volcano plot of the differentially expressed genes between sh-NNMT-HESC and sh-NC-HESC with ERBB4 highlighted.**

**Figure S7.**

**S7A**


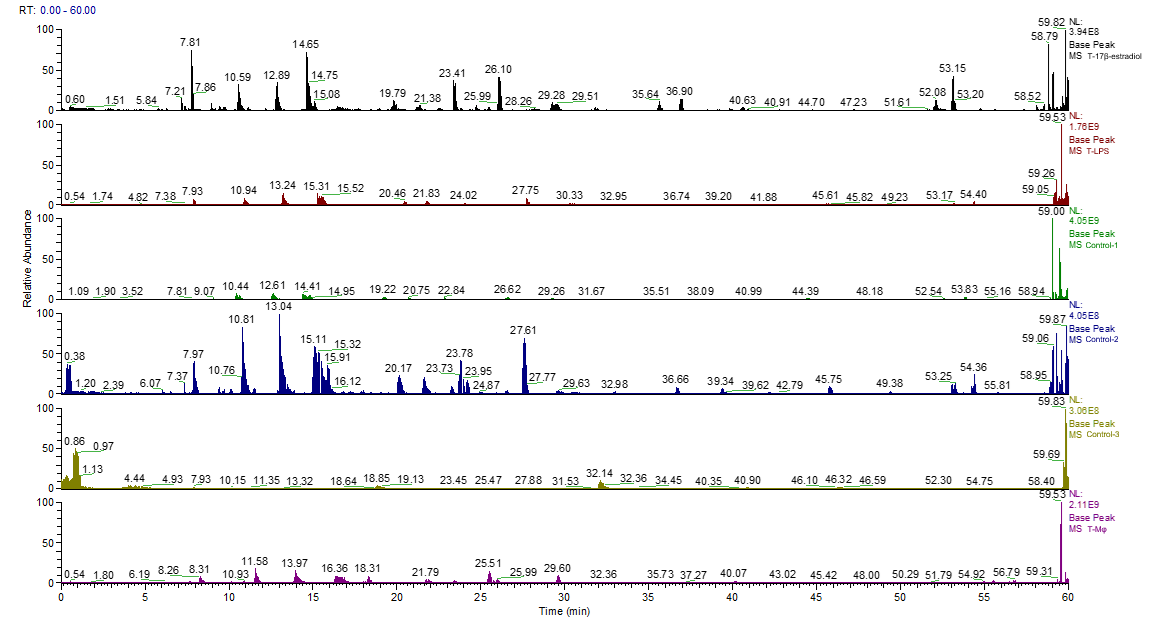


**S7B**


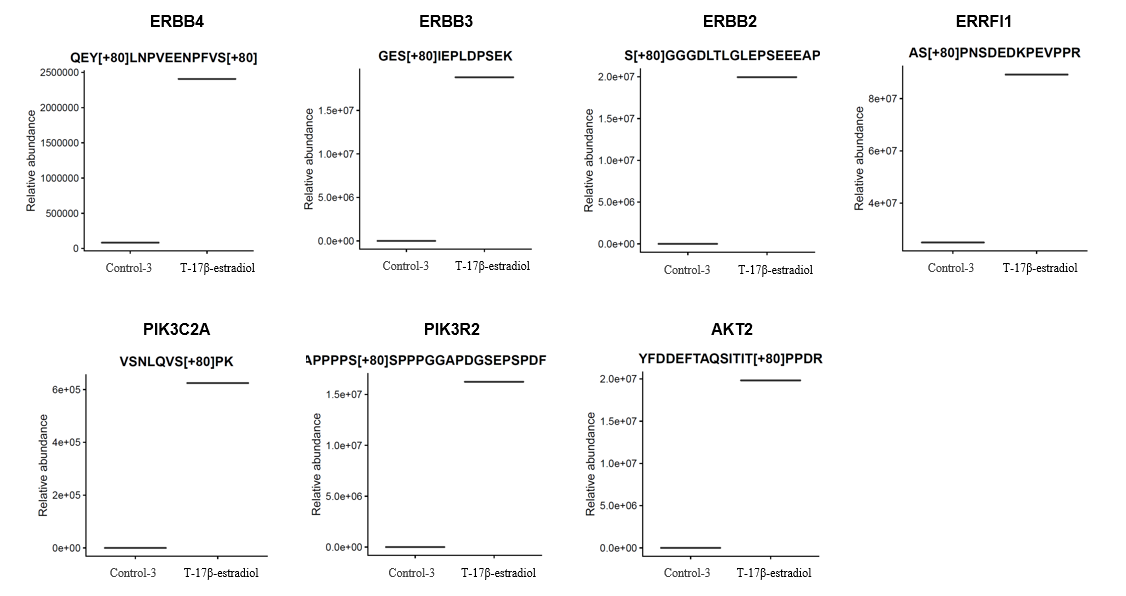


**S7C**


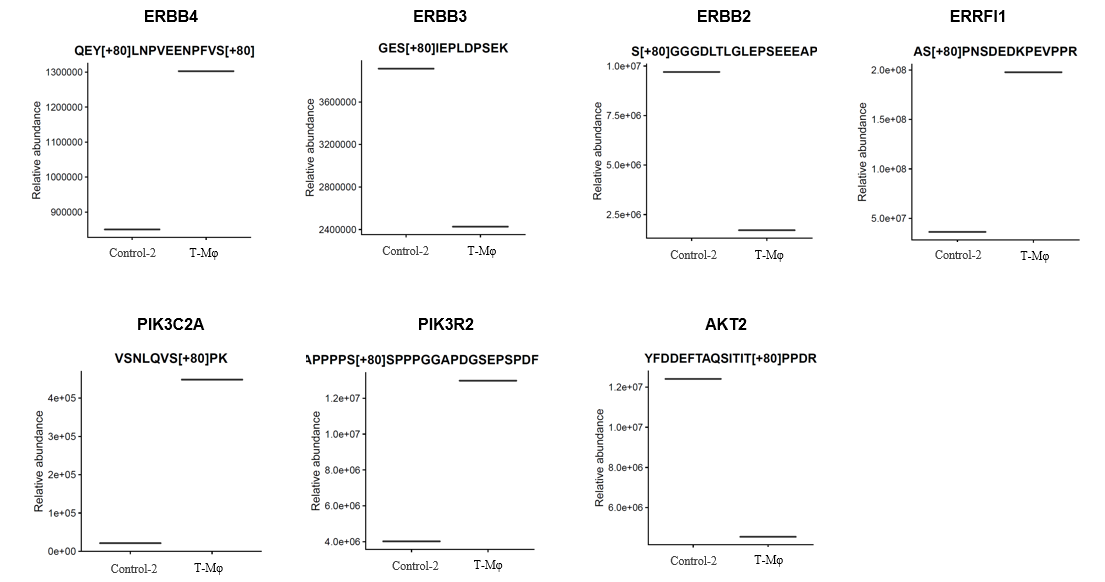


**Figure S7.** **The Parallel Reaction Monitoring (PRM) targeted proteomics and analysis.** (A) The basepeak diagram of the samples. The target peptides were detected in HESCs treated with macrophage-conditioned medium (T-Mφ) and 100 nM 17β-estradiol (T-17β-estradiol), with the LPS-treated group (T-LPS) used as a positive control. (B) Quantitative analysis of target peptides in the T-17β-estradiol and control groups. (C) Quantitative analysis of target peptides in the T-Mφ and control groups. ERBB4/3/2, erb-b2 receptor tyrosine kinase 4/3/2. ERRFI1, ERBB receptor feedback inhibitor 1. PIK3C2A, phosphatidylinositol-4-phosphate 3-kinase catalytic subunit type 2 alpha. PIK3R2, phosphoinositide-3-kinase regulatory subunit 2. AKT2, AKT serine/threonine kinase 2.
