# Supplementary material for: Overexpressed nicotinamide N‑methyltransferase in endometrial stromal cells induced by macrophages and estradiol contributes to cell proliferation in endometriosis
Source: Cell Death Discov. 2024 Nov 3;10:463. doi: 10.1038/s41420-024-02229-3 (PMC11532478; doi:10.1038/s41420-024-02229-3)
Supplement: Supplementary file 2 — Original western blots [file 41420_2024_2229_MOESM2_ESM.docx]

**Figure 1H**

VINCULIN 1


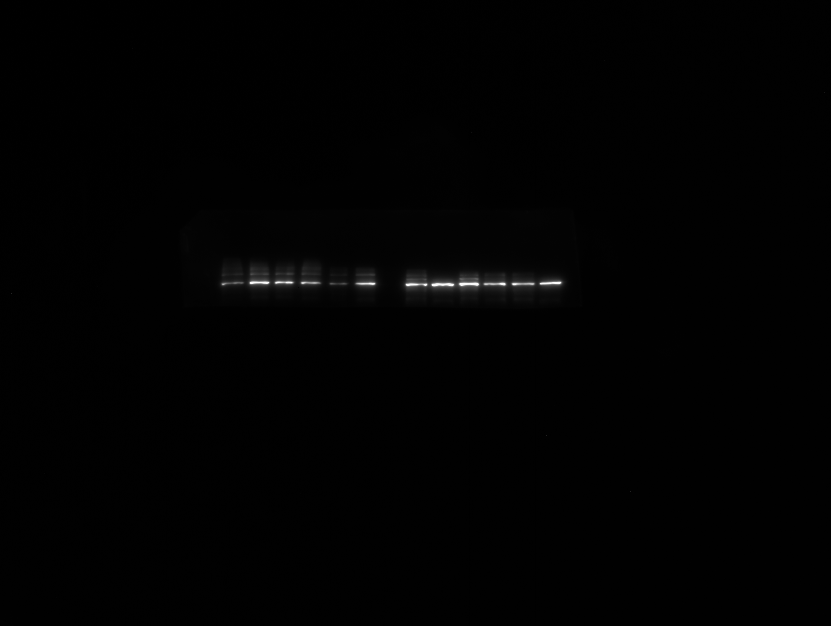


NNMT 1


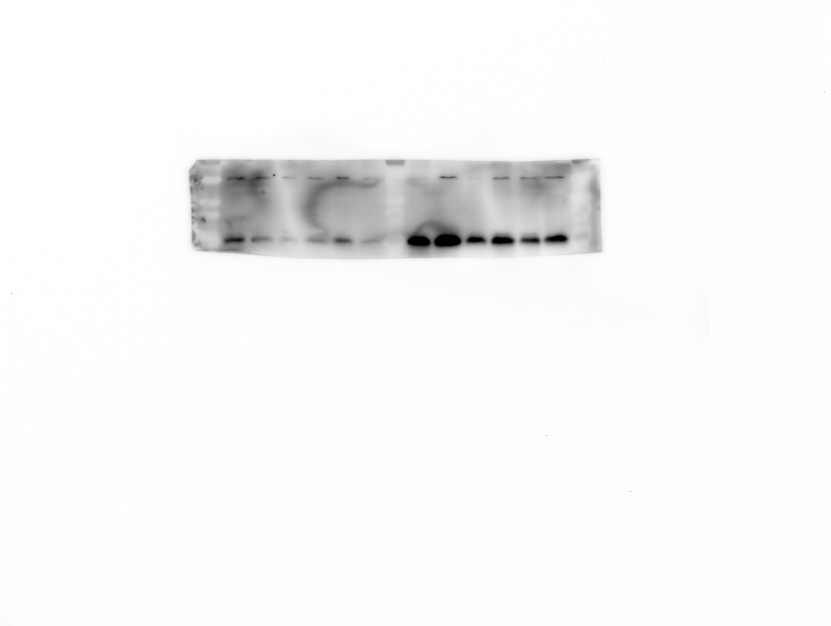


VINCULIN 2


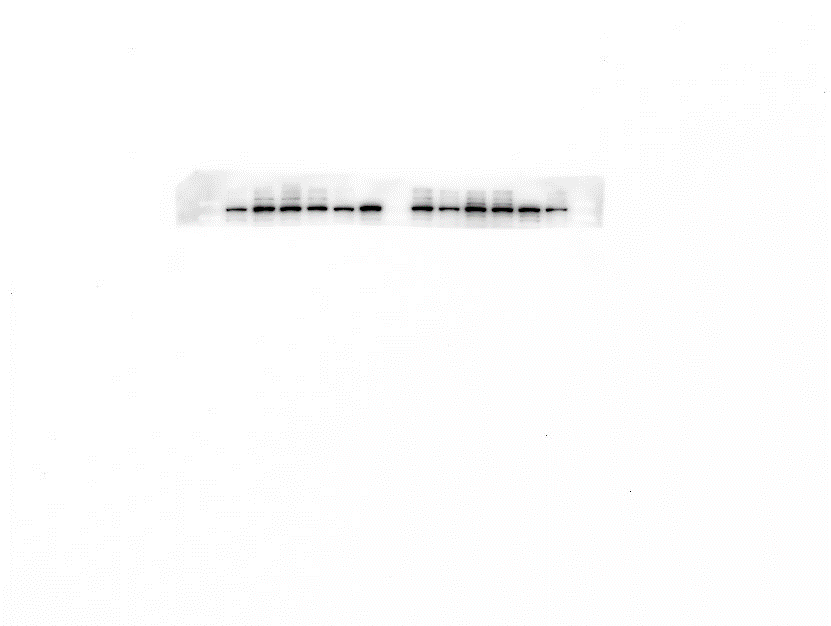


NNMT 2


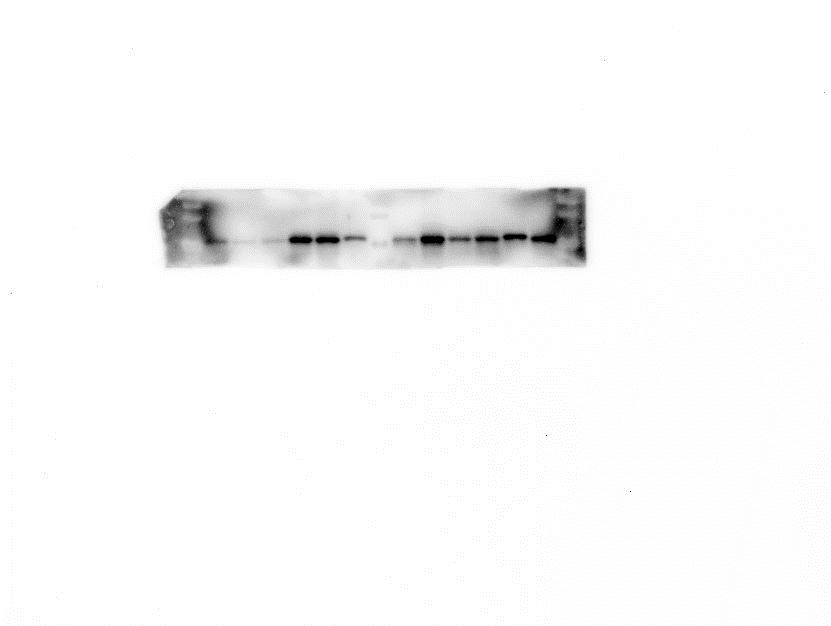


**Figure 2D**

VINCULIN 1


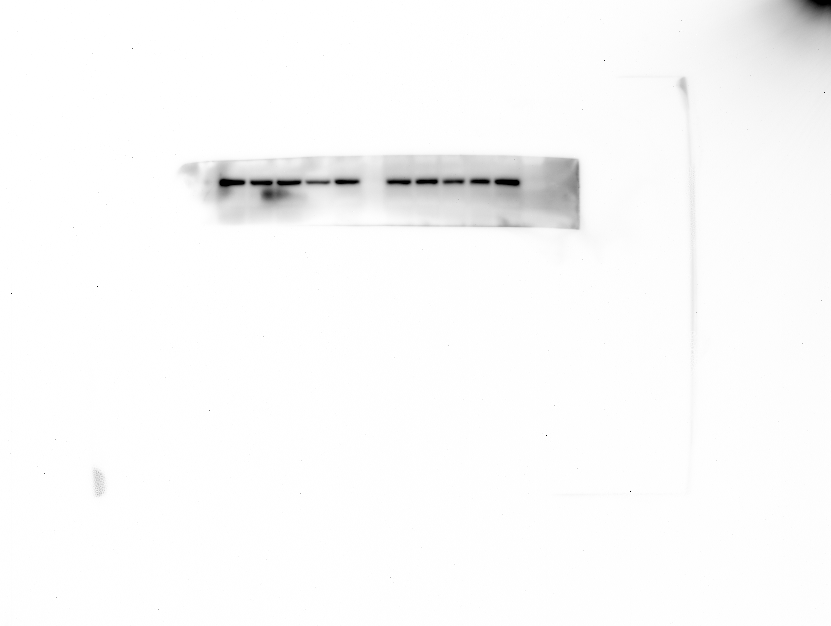


NNMT 1


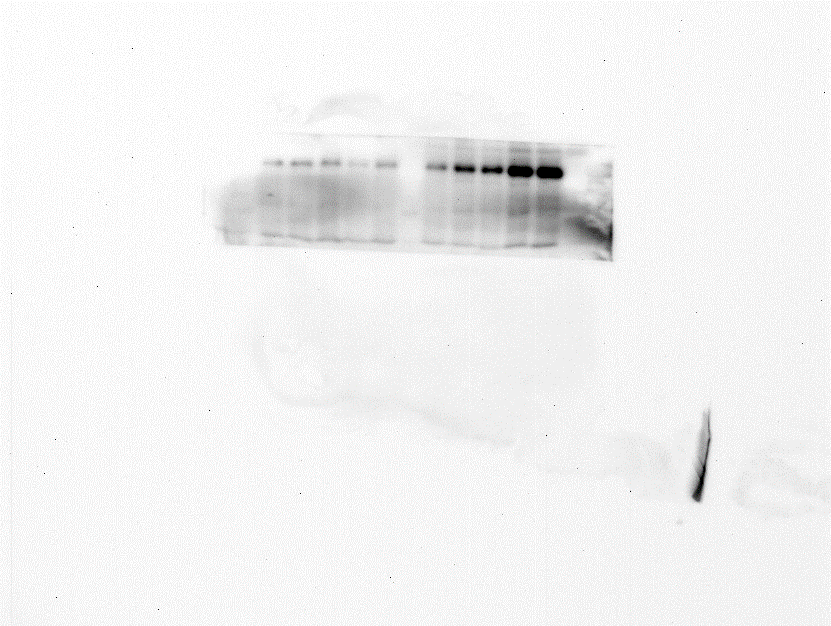


VINCULIN 2


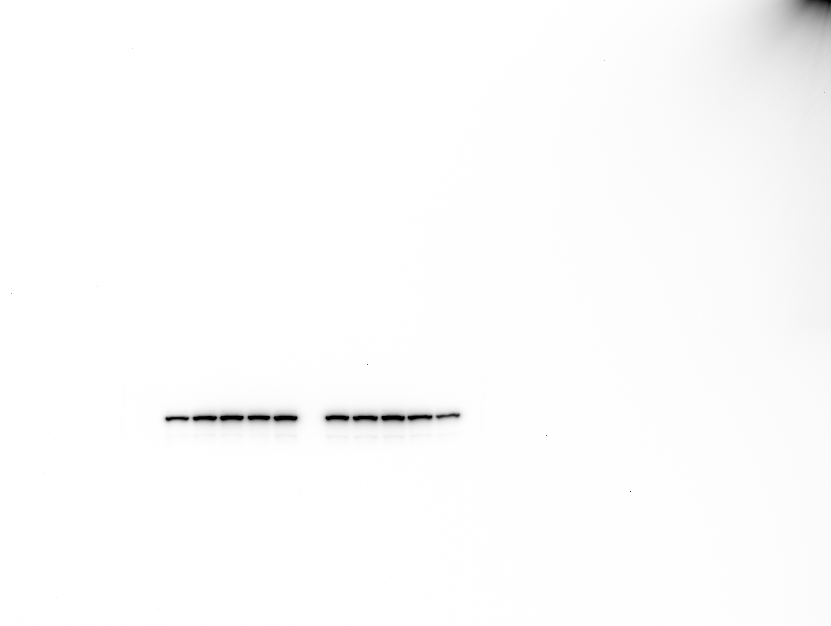


NNMT 2


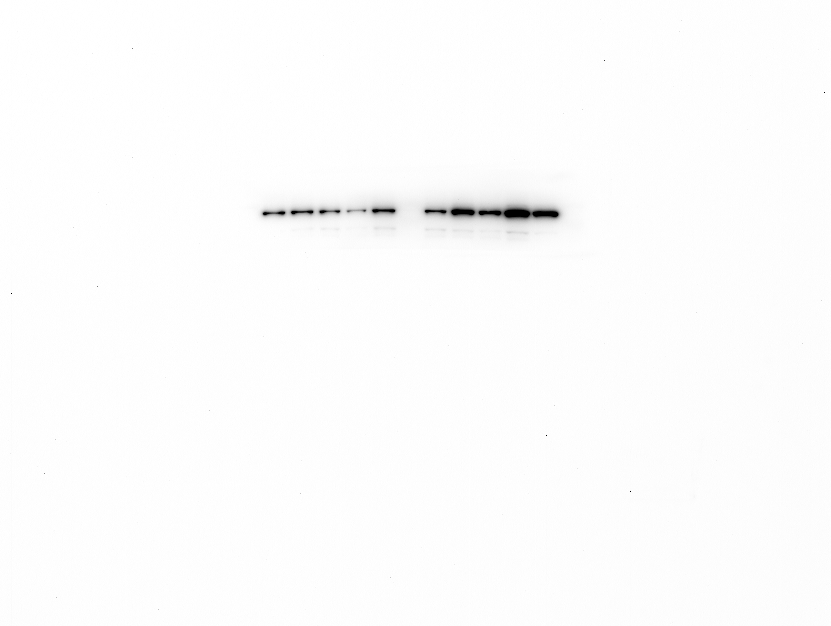


**Figure 4A**

α-TUBULIN


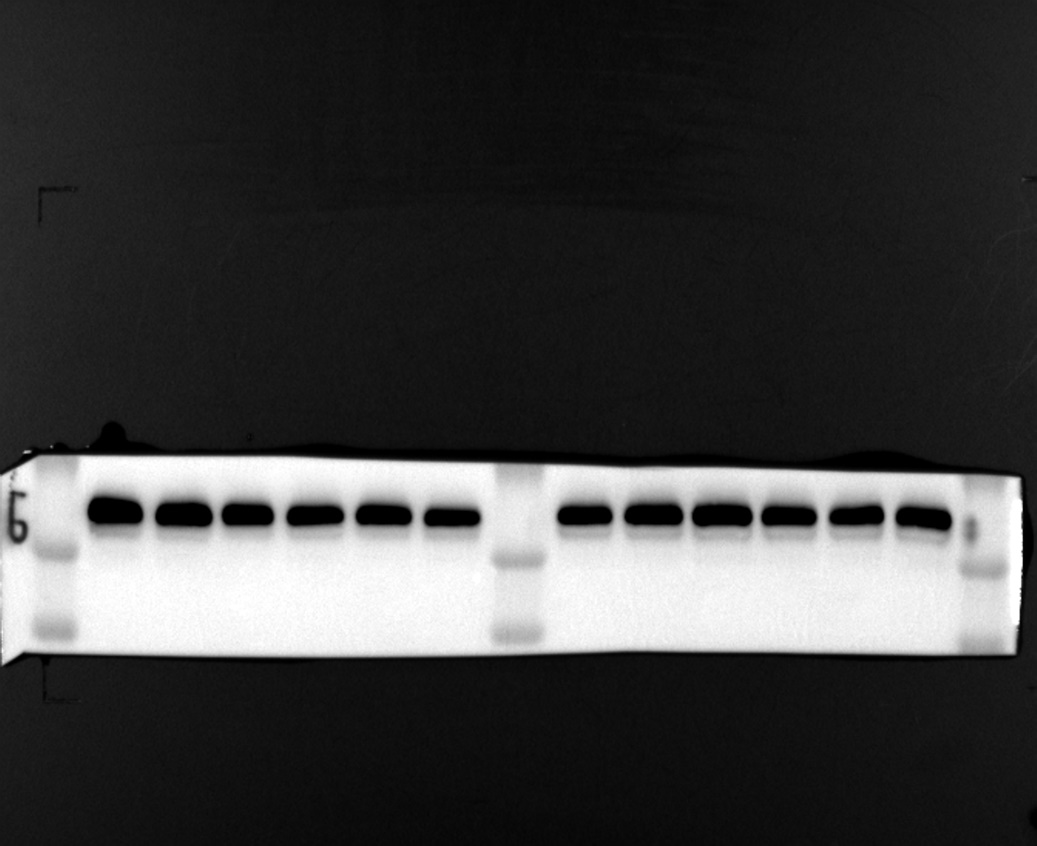


NNMT


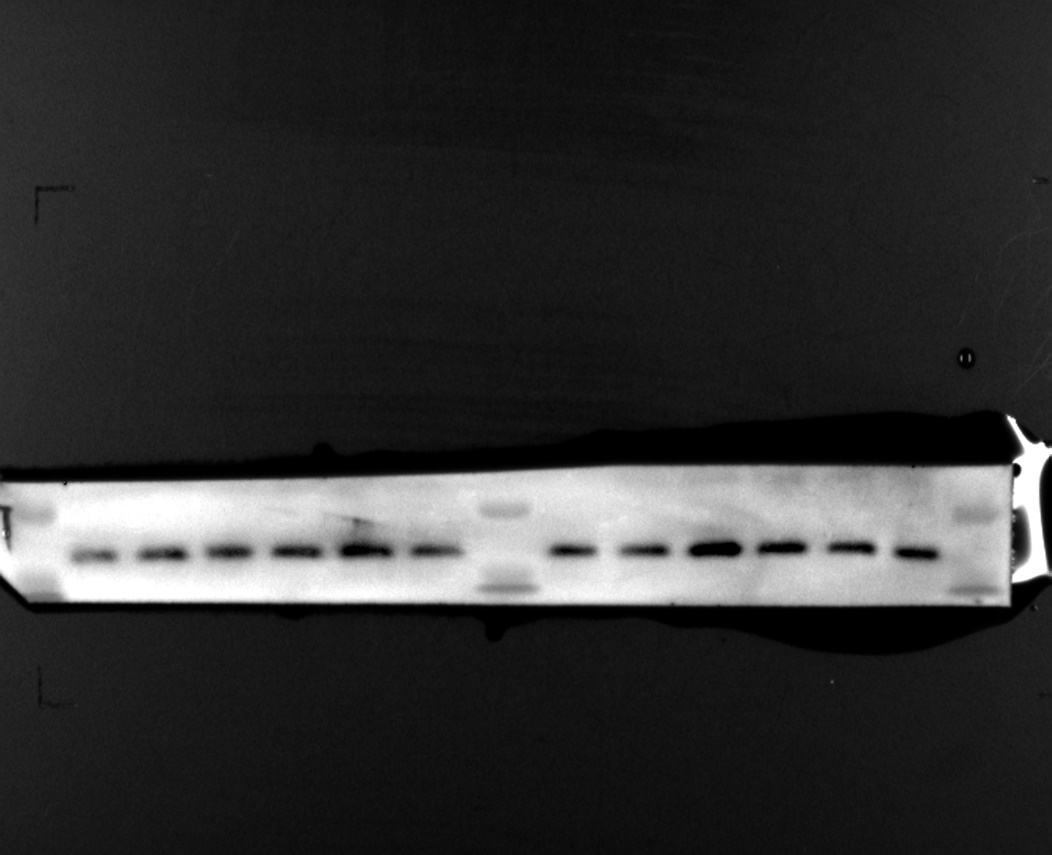


**Figure 4C**

VINCULIN 1


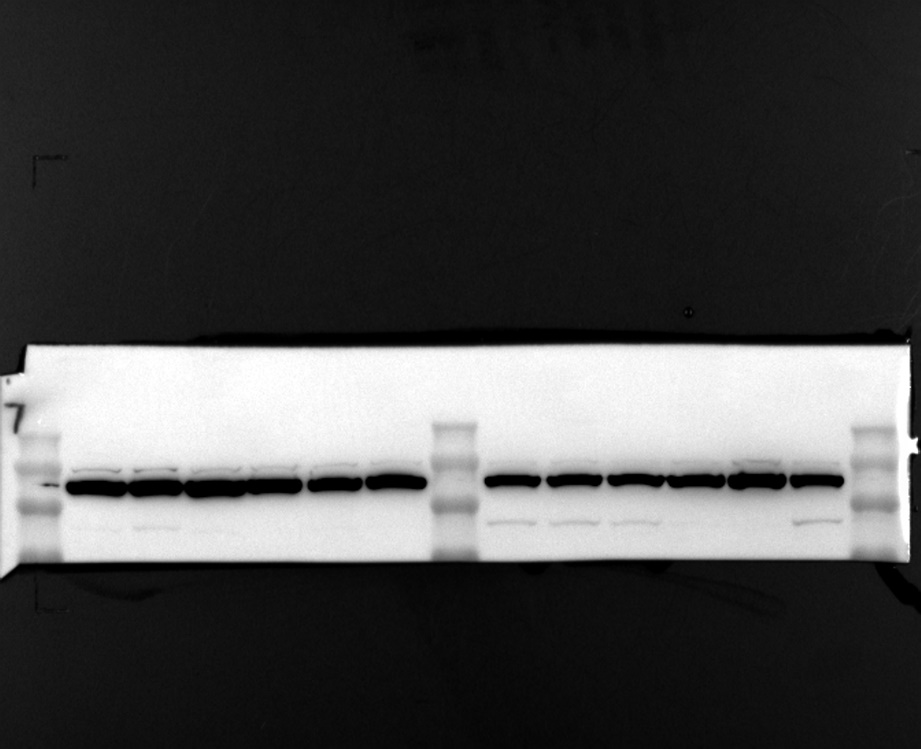


NNMT 1


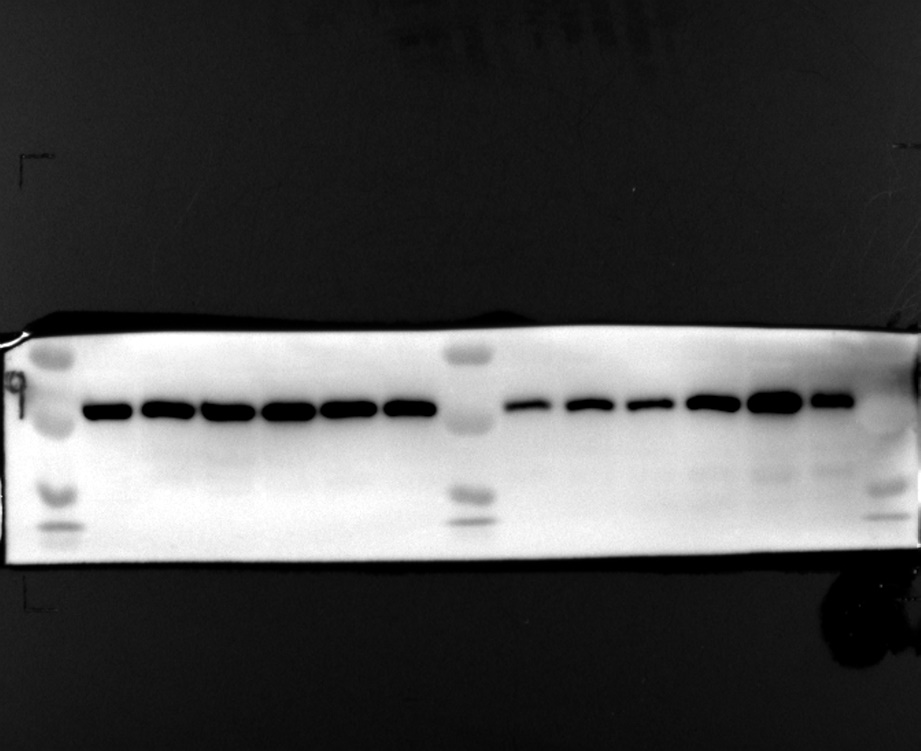


VINCULIN 2 3


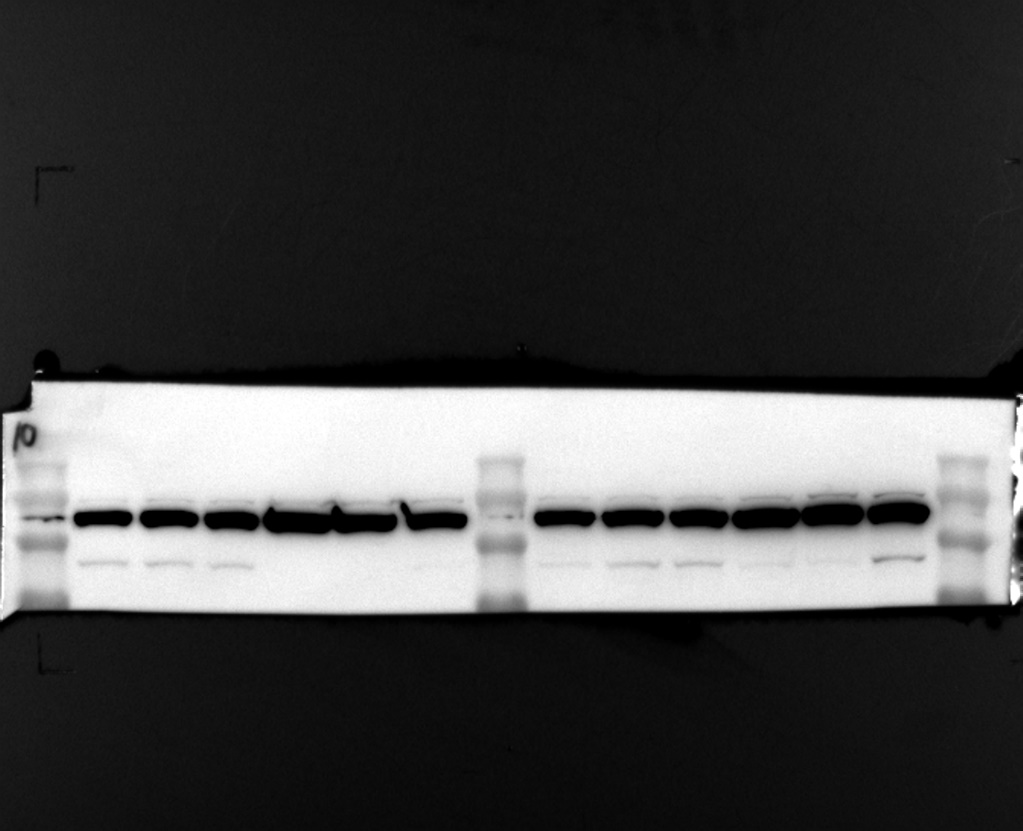


NNMT 2 3


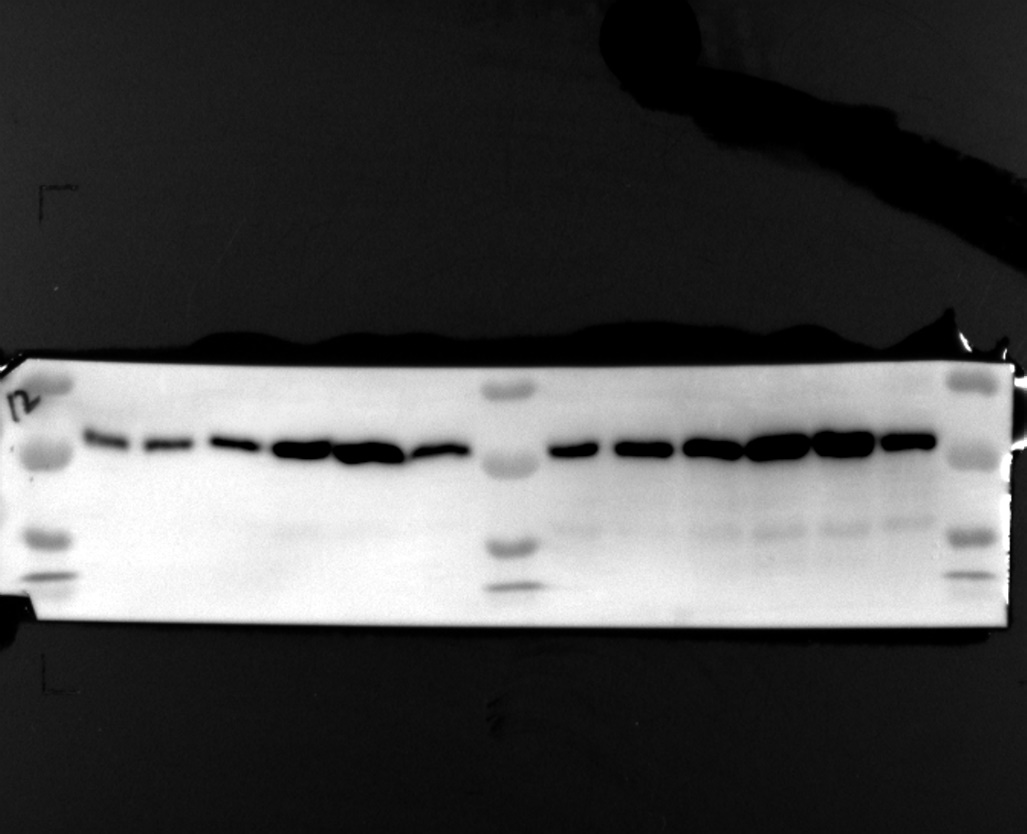


**Figure 5B**

α-TUBULIN


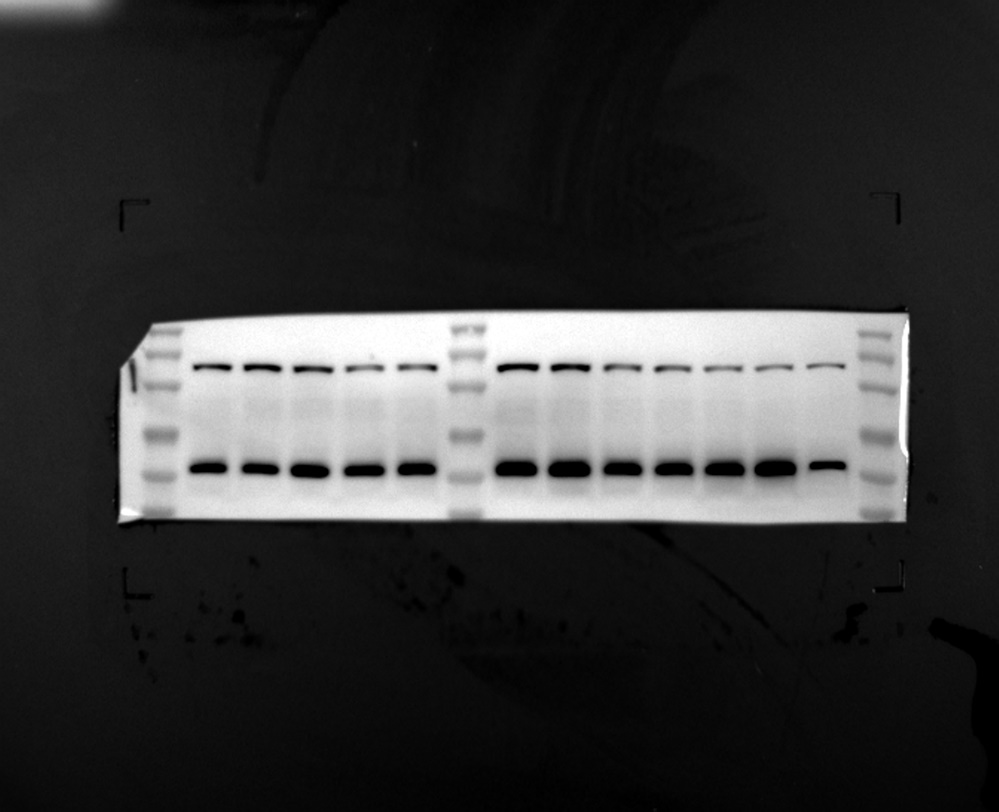


NNMT


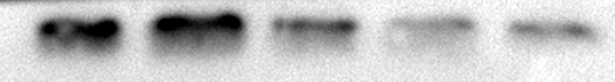


**Figure 6B**

**β-ACTIN**





NNMT





ERBB4





p-ERBB4





PI3K





p-PI3K





AKT





p-AKT


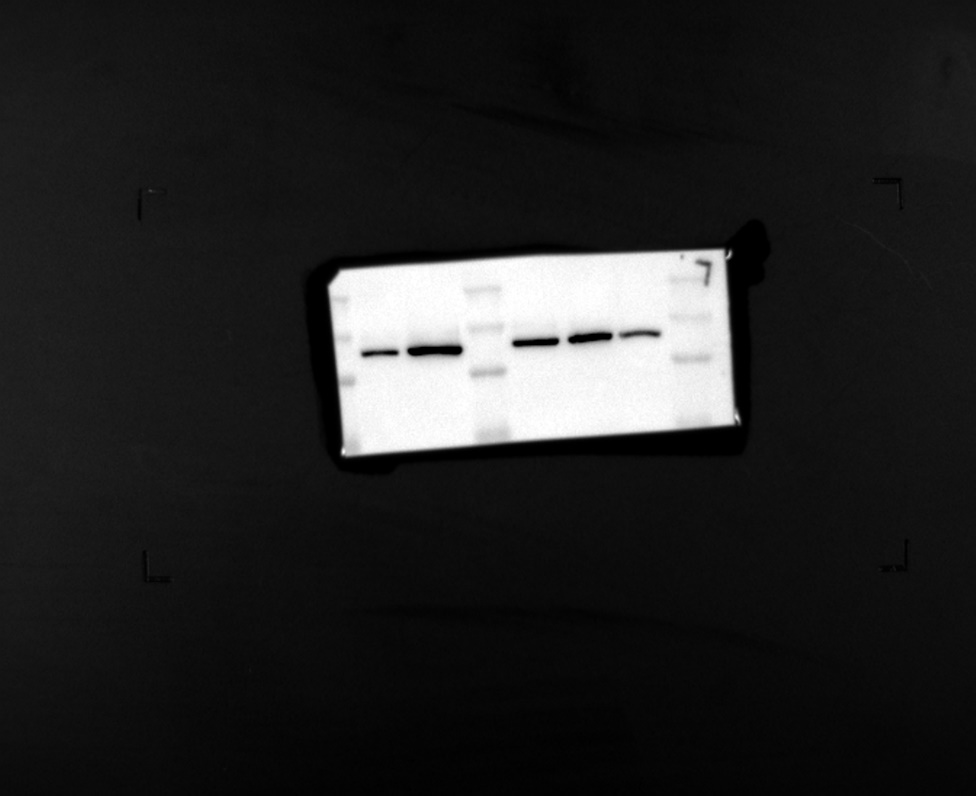


**Figure 6C**

**β-ACTIN**





NNMT





ERBB4





p-ERBB4


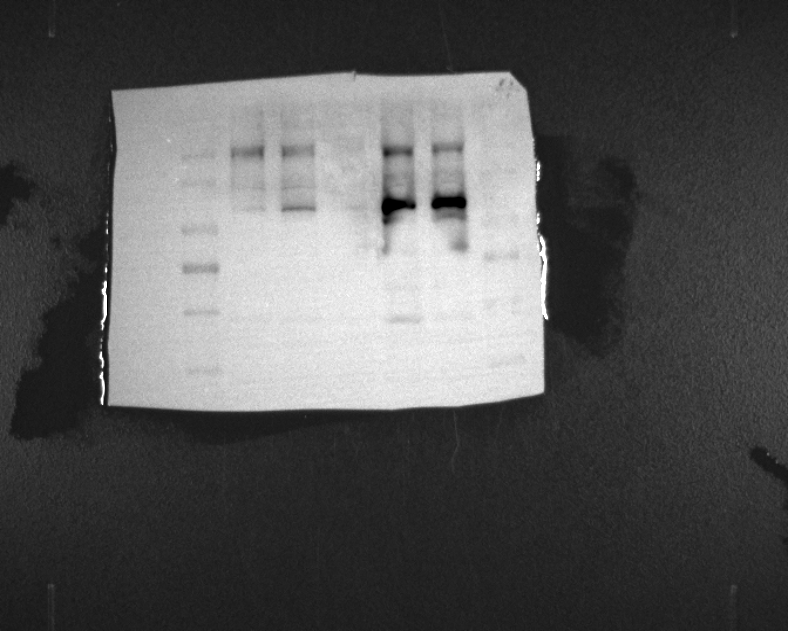


PI3K





p-PI3K





AKT





p-AKT
